# SMEs as Agents of Social Cohesion: Opportunities, Challenges, and Conceptual Framework

**DOI:** 10.12688/f1000research.183208.1

**Published:** 2026-06-30

**Authors:** Malek Al-Edenat, Nayel AL Hawamdeh

**Affiliations:** 1 Business, Tafila Technical University College of Business, At Tafilah, `Amman, Jordan, Tafilah Governorate, 66110, Jordan; 2 Business, Tafila Technical University College of Business, At Tafilah, `Amman, Jordan, Tafilah Governorate, 66110, Jordan

**Keywords:** Social Cohesion, Community Businesses, Community Development, Community Innovation, Social Enterprise

## Abstract

Small and medium enterprises (SMEs) are widely acknowledged as key contributors to inclusive and sustainable development. However, their role as agents of social cohesion remains conceptually fragmented and insufficiently developed. This paper addresses this gap by proposing an integrated conceptual framework explaining how SMEs foster social cohesion through local embeddedness, employment practices, and stakeholder engagement. Drawing on interdisciplinary perspectives from entrepreneurship, social capital, and corporate social responsibility, the study identifies key opportunities, including job creation, social inclusion, and community-driven innovation. It also outlines critical constraints, such as limited resources, weak institutional environments, and the prevalence of informal practices. By synthesizing these opportunities and challenges, the paper advances existing literature and offers a coherent analytical model supported by research propositions for future empirical studies. The study provides valuable insights for policymakers and practitioners aiming to position SMEs as effective drivers of social cohesion, particularly within emerging and resource-constrained economies.

## 1. Introduction

In a progressively linked world, social cohesiveness within businesses has become crucial for promoting cooperation and creativity, especially in small and medium enterprises (SMEs). These businesses frequently encounter distinct factors that affect their ability to foster a unified culture, such as resource constraints, employee demographics, and differing stakeholder expectations ( Turner, 2001). As SMEs confront these difficulties, they simultaneously have unprecedented opportunities to utilize their agility and cohesive settings to strengthen social connections and trust ( Lukito-Budi et al., 2023). The interaction between these possibilities and difficulties influences the internal structure of these organizations and has wider ramifications for their sustainability and community impact ( Lee, 2009).

Social cohesion refers to the connections that integrate individuals within a community, fostering mutual respect, trust, and a collective sense of belonging ( Delhey & Dragolov, 2016). Additionally, social cohesion is vital for enhancing collaboration and innovation within small and medium enterprises (SMEs). Furthermore, employees’ connections with each other and their alignment with the organizational mission positively correlate with increased engagement and productivity levels ( Oketch, 2004; Rumasukun & Noch, 2023). Social cohesion can mitigate conflicts and improve communication, thus creating a positive work environment essential for growth and development ( Aall & Crocker, 2019). Therefore, the interaction between social cohesion and organizational success highlights the importance of SMEs prioritizing the cultivation of inclusive and supportive social networks to navigate their unique challenges ( Loukopoulos et al., 2024). For instance, the Danish model shows that higher social solidarity is linked to economic stability, suggesting that cohesive organizations can effectively address external challenges. The association between social cohesion and organizational success emphasizes SMEs’ need to develop inclusive and supportive social networks to tackle their specific difficulties ( Rumasukun & Noch, 2023).

From a different point of view, social cohesion is characterized by a collective sense of belonging within a group, the establishment of bonds and connectedness among community members, and the facilitation of positive social interactions ( Kedi et al., 2024a). Groups exhibit cohesion when group-level conditions foster positive attitudes and behaviors among members, and when interpersonal interactions sustain these conditions ( Friedkin, 2004). It has been claimed that responsible actions enhance a company’s reputation. Corporate contributions to society are advantageous; hence, fostering social cohesiveness is in the company’s best interest ( Koonce, 2011).

This paper will explore the complex dynamics of social cohesiveness in SMEs, shedding light on opportunities for SMEs to enhance social cohesiveness and tactics to surmount current challenges while using the inherent advantages of smaller enterprises. Ultimately, comprehending this dichotomy is crucial for effectively promoting social cohesiveness within the framework of SMEs.

## 2. Opportunities for enhancing social cohesion

### 2.1 Innovation and creativity

A notable possibility for enhancing social cohesion in SMEs is in the capacity for innovative collaborations that utilize local resources and promote community involvement. Such programs’ efficacy frequently relies on forming networks that promote contact among varied cultural and socioeconomic groups. A research on the Danish model demonstrates that a high level of income equality and a cooperative approach to labor markets foster an atmosphere that promotes social cohesion, underscoring the significance of shared objectives and mutual support ( Lundvall, 2007). For example, promotional initiatives in creative tourism may fortify relationships among community members and elevate local identity, as demonstrated by studies advocating for new frameworks to facilitate interactions between cultural and tourist sectors ( Vieira et al., 2020). By adopting these collaboration opportunities, SMEs may foster a more inclusive and coherent social structure ( Zahoor et al., 2020).

In SMEs’ context, improved employee cooperation and teamwork are essential for promoting social cohesion and enhancing overall performance. The transition to remote work and the use of virtual teams have presented both possibilities and obstacles. Virtual teams enable enterprises to aggregate varied talents and experiences without temporal and spatial limitations, thereby improving problem-solving abilities and fostering innovation ( Tang et al., 2020). Successful collaboration depends significantly on efficient communication, team resilience, and recognizing individual diversity; these components are crucial for establishing trust and fostering cohesive work dynamics ( Gucciardi et al., 2018). Leaders must proactively inspire team members, delineate distinct responsibilities, and promote mutual comprehension to surmount distance challenges. Consequently, although collaboration has considerable potential for SMEs, it requires meticulous oversight to preserve and enhance social cohesiveness ( Minhas & Sindakis, 2022).

### 2.2 Flexibility and agility

SMEs can exhibit greater agility in addressing social issues and performing programs that foster social cohesion. They can rapidly modify to evolving circumstances, explore innovative strategies, and render choices more promptly than prominent entities. An SME can swiftly initiate a community outreach campaign to address a local social issue, utilizing its adaptability to customize the program according to the community’s requirements ( Koçyiğit & Akkaya, 2020).

This is true compared with larger enterprises. However, this is contingent upon their strategies, culture, and leadership. Their diminutive size, streamlined structures, and stronger client interactions frequently enable them to react more swiftly to changes. In fact, SMEs responded faster to crises ( Klein & Todesco, 2021).

Moreover, SMEs benefit from various factors that make them more agile and flexible. For example, and not limited, first, a streamlined organizational structure characterized by fewer hierarchical layers facilitates expedited decision-making (e.g., eliminating protracted board approvals). At the same time, personnel frequently assume numerous roles, promoting cross-functional collaboration ( A. D. Zaridis & Mousiolis, 2014). Secondly, the proximity of customer interactions enables SMEs to engage directly with clients, facilitating rapid awareness of market changes and the customization of solutions. Upon seeing client demand, a small bakery may transition to gluten-free offerings ( Konsti-Laakso et al., 2012). Third, adaptable processes that alleviate the burdens imposed by stiff legacy systems or bureaucratic procedures on SMEs. SMEs can swiftly change suppliers amid a supply chain problem ( Nakandala et al., 2024). Fourth is entrepreneurial culture, in which founders or owners frequently foster innovation and experimentation. This is evident as a technology startup transitions from a B2C model to a B2B one, following market viability assessments ( Shaw, 2014). Fifth, the risk of change is diminished. A reduced size entails fewer parties to persuade and less difficulty in executing modifications. A 10-person marketing agency implements AI techniques more rapidly than a multinational corporation ( Falkner & Hiebl, 2015).

### 2.3 Robust community connections

SMEs frequently cultivate more robust and personalized connections with their communities. This establishes trust and promotes deeper engagement, cultivating a sense of belonging and inclusion. In that, a local organization can utilize its robust connections with community leaders to orchestrate activities that unify various groups and foster social cohesion ( Spence & Schmidpeter, 2003). In fact, SMEs are frequently referred to as ‘employment engines’ and are seen as the foundation of economic development ( Muriithi, 2017; Robu, 2013; Surya et al., 2021). To serve this crucial economic function, SMEs must adjust to many internal and external circumstances, including their geographical location. In contrast to urban environments, SMEs and their owner-managers tend to be more embedded within their rural communities and society. Moreover, SMEs are profoundly impacted and molded by the contexts in which they function, whether in rural or urban settings ( Child et al., 2022; Korsgaard & Tanvig, 2015; Williamson et al., 2006).

Consequently, their sense of responsibility for their regions and society tends to be greater, motivating SMEs in rural areas to influence their future, generate new employment opportunities, pursue community advantages, and encourage innovation ( Cravo et al., 2012; Lang et al., 2014). These regions can also pose exceptional historical, cultural, or physical resources ( Korsgaard et al., 2015; Manzoor et al., 2021), as a result expanding opportunities for SMEs. Furthermore, the strong connection between SMEs and the local community is reinforced by the fact that many SMEs in rural regions are family-owned. Because the family that runs the business lives in the same spatial environment where their firm functions, having a good reputation in the community is crucial for both the business and their personal life ( Banki & Ismail, 2015; Larsson et al., 2003). That is, rural communities are considered close-knit. This embeddedness appears to provide an additional advantage to SMEs. Studies demonstrate that SMEs in rural regions possess a more intuitive comprehension of their area’s physical, historical, and cultural composition ( Beckmann et al., 2023; Korsgaard & Tanvig, 2015).

### 2.4 Localized impact

SMEs may concentrate on hyper-local concerns and customize their activities to address the distinct requirements of their community. This hyper-local strategy can result in more significant and pertinent social cohesion initiatives, wherein a community-based organization can develop a program that targets the unique social issues encountered by a particular neighborhood, such as intergenerational differences or ethnic conflicts ( Harte et al., 2016; Makkonen & Leick, 2020; McCann & Ortega-Argilés, 2016). Even though SMEs located in metropolitan areas have an advantage over those in regional areas ( Freeman et al., 2012), however, localization catalyzes brand expansion and facilitates consumer access to products. In addition, operating within local and small communities enables SMEs to better understand the requirements of the individuals in these communities ( Chhibber & Chadha, 2020).

More importantly, the location of SMEs is crucial in promoting social cohesion by meeting community-specific requirements, overcoming disparities, and generating collective economic and cultural value. Meanwhile, SMEs inside communities immediately invigorate local economies by generating employment, especially for marginalized demographics such as women and youth. In Northern Uganda, women-led SMEs utilized social cohesiveness as “social collateral” to get microfinance, facilitating the reconstruction of livelihoods in post-war communities where physical collateral had been obliterated ( Bongomin et al., 2020). In Jordan, SMEs constitute over 90% of private enterprises, employing more than half of the workforce, enhancing economic stability and mitigating disenfranchisement ( Etihad, 2023). Moreover, Local SMEs frequently partner with grassroots organizations to tackle urgent societal challenges, including education and infrastructure. These collaborations foster trust and reciprocal support, shown by efforts in which SMEs collaborate with non-profits to develop local parks or schools. These observable, concrete results strengthen community ties and motivate collaborative efforts. Moreover, staff participation through SME-led CSR activities enhances morale and fortifies connections between firms and the community ( Smith et al., 2022).

Further, SMEs situated within varied communities serve as centers for cultural exchange. They advance sustainability while honoring worldwide fashion history and cultivating a respect for cultural variety. Likewise, several enterprises provide common areas facilitating connections among individuals from diverse origins, dismantling preconceptions and fostering mutual respect. This cultural interchange fortifies a collective identity and diminishes social discord ( Joseph & Van Buren, 2022). In post-conflict regions, SMEs serve as foundational elements for the reconstruction of social networks. Women’s micro-enterprises in that region depend on social cohesiveness to get loans and maintain operations, illustrating how localized SMEs may supplant disjointed systems with collaborative structures. These firms offer economic stability and emotional support, which are essential for rehabilitating fractured communities ( Djip, 2014). Finally, such kinds of SMEs frequently emphasize environmental practices, including the procurement of materials locally to minimize carbon footprints, such as those providing farm-to-table enterprise, promoting organic agriculture and community self-sufficiency, and harmonizing economic development with environmental stewardship. These efforts cultivate communal pride in environmental care, bringing communities together around common ideals ( Agan et al., 2013).

### 2.5 Customized engagement

SMEs may interact with individuals in a more personalized manner, fostering deeper ties and more significant engagements. This can foster a sense of community and belonging. This means SMEs utilize tailored engagement tactics to promote social cohesion by establishing significant relationships with their target consumers, connecting brand values with community needs, and developing inclusive digital ecosystems. A small advocacy organization can establish one-on-one mentorship programs that connect persons from diverse backgrounds, promoting mutual understanding and respect ( Iyelolu et al., 2024; Wee & Chua, 2013). Recently, SMEs have used digitalization and artificial intelligence to customize and personalize engagement. In contrast to international or multinational firms, SMEs, somewhat, deal with small communities, allowing the use of artificial intelligence-powered instruments for the analysis of consumer data, which, in turn, facilitates customized interactions that align with individual tastes and behaviors ( Telukdarie et al., 2022; Kedi et al., 2024b), especially, in emerging economies ( Abrokwah-Larbi & Awuku-Larbi, 2024).

In addition, social media is a principal medium for SMEs to engage audiences in ways that foster inclusivity, such as user-generated content (UGC) campaigns that motivate customers to share experiences and cultivate peer-to-peer connections. For example, highlighting customer success stories or conducting interactive polls creates a participatory community ( Ohara et al., 2024; Qalati et al., 2021, 2022). Likewise, SMEs customize content for specific platforms like TikTok (targeting younger demographics) or LinkedIn (for B2B networking), ensuring relevance and inclusivity ( Kedi et al., 2024b; Kraus et al., 2019). At the same time, real-time interaction involves responding to comments, messages, and forum discussions (e.g., Reddit, Quora), thereby humanizing the brand and enhancing communal trust ( Huang & Liu, 2021). As a result, these initiatives convert passive audiences into active participants, reinforcing social cohesion through shared narratives ( Belair-Gagnon et al., 2019).

Another benefit for SMEs in fostering social cohesion is using social-customer relationship management for collaborative innovation ( Hassan et al., 2019). This allows SMEs to engage customers in co-creation processes, such as feedback loops, which actively seek customer input through surveys or social media polls. This practice ensures that customer perspectives influence product development, cultivating a sense of ownership ( Bodó et al., 2019). Co-creation initiatives invite customers to contribute ideas for new innovative goods and services to enhance emotional engagement with the brand ( Fernandes & Remelhe, 2016). Consolidated customer profiles integrating data from several channels enable SMEs to provide consistent, personalized experiences, enhancing trust. This cooperative strategy connects businesses and communities, fostering reciprocal development ( Hayes & Kelliher, 2024).

Ultimately reconciling automation with human sensitivity. Although automation improves efficiency, SMEs acknowledge the indispensable contribution of human insight in fostering genuine engagement through AI-human collaboration, wherein social media managers employ AI tools for routine tasks while personally addressing sensitive interactions with emotionally intelligent avatars ( La Sala et al., 2024). Sentiment analysis methods assist AI systems in identifying irritation or happiness, allowing sympathetic reactions that reflect human sensibility ( Egger et al., 2019). Localized content in which SMEs modify automated material to embody local subtleties, guaranteeing inclusion ( Raji et al., 2024). Consequently, this equilibrium guarantees that technology augments, rather than supplants, the human element, which is essential for social cohesiveness.

In conclusion, SMEs have the potential to enhance social cohesion within the communities they operate in through five opportunities: enhanced collaboration and teamwork among employees, flexibility and agility, robust community connections, localized impact, customized engagement, and innovation and creativity. Figure 1 illustrates the above-mentioned opportunities for SMEs in their endeavor to enhance social cohesion.

**Figure 1.**
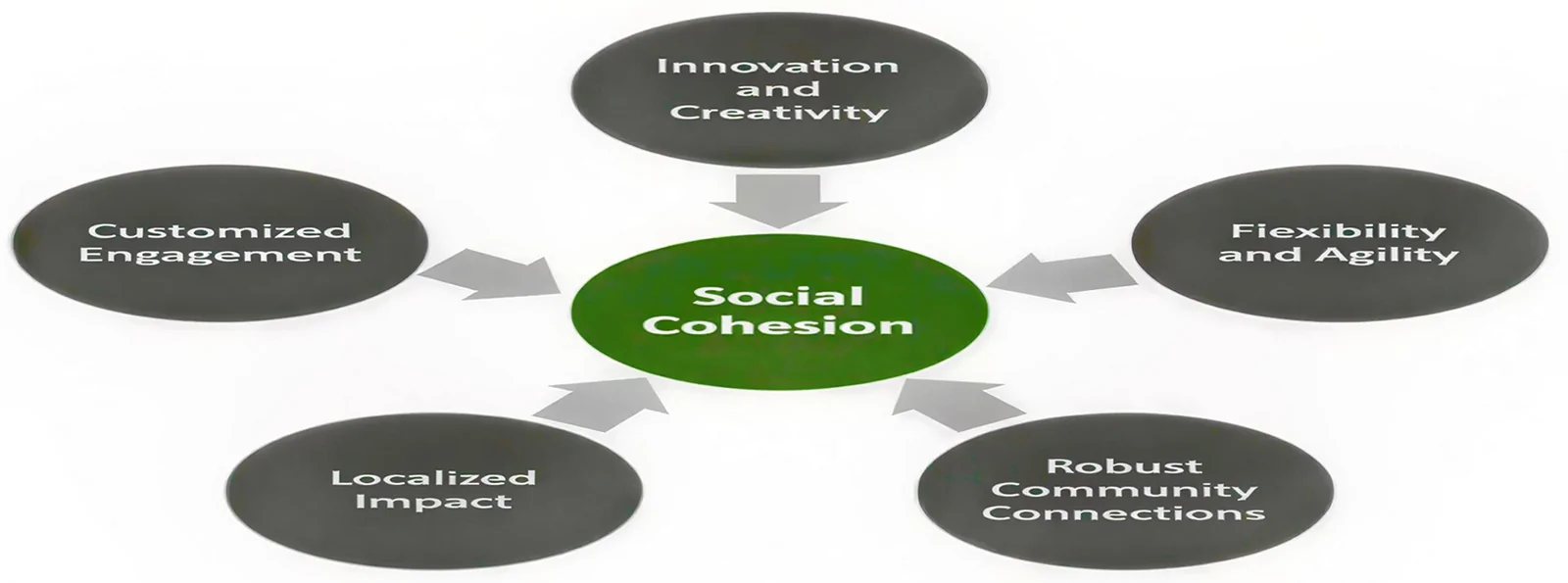
SMEs’ Five opportunities to enhance social cohesion.

## 3. Challenges facing SMEs in enhancing social cohesion

In this section, we will shed light on the challenges, or in other words, determinants, that could hinder SMEs in their pursuit of achieving the best level of social cohesion. These challenges will be critically discussed to shape understandable and clear perceptions for SMEs about these challenges that may prevent these firms from achieving a high level of social cohesion. In addition, these perceptions may provide scope for drawing a roadmap that may benefit these companies to overcome these challenges.

Enhancing social cohesiveness in SMEs encounters several hurdles stemming from both internal dynamics and external forces. A significant challenge is the intrinsic variety inside organizations, which, although advantageous for creativity, may also result in misunderstandings and disputes among individuals from varied backgrounds ( Yahaya & Nadarajah, 2023). Furthermore, as mentioned above, the Danish model emphasizes that the economic background indicates that although high-income equality and stability promote social cohesion, economic development stagnation may jeopardize these circumstances’ maintenance ( Lundvall, 2007).

### 3.1 Limited resources

Typically, SMEs have limited financial, human, material, and knowledge resources, which can hinder their ability to implement and sustain social cohesion programs, especially in developing countries ( Prasanna et al., 2019). For example, a smaller firm might struggle to fund a large-scale community event that brings together diverse groups, thus limiting its impact ( Blackburn & Ram, 2006). In addition, SMEs often operate on limited budgets, making it challenging to allocate resources for corporate social responsibility as well as the lack of expertise in measuring corporate social responsibility initiatives that foster social cohesion, such as community engagement programs, diversity training, or partnerships with local organizations ( Jenkins, 2006, 2009; Sweeney, 2007). Frequently, SMEs are unable to invest in long-term projects. Whereas promoting social cohesion often necessitates ongoing efforts and long-term investments, which SMEs might find difficult to afford due to their limited financial resources ( Nicolas, 2022).

Moreover, human resource constraints hinder SMEs from attaining greater social cohesiveness. This results in a deficiency of committed personnel. Due to the often smaller teams in SMEs and the tendency for individuals to assume several jobs ( Desouza & Awazu, 2006), there is a little capacity for staff in SMEs to concentrate on projects that need special skills in interacting with customers through different channels such as social media ( Ramsay, 2010), the lack of these skills that resulted from limited human resources might negatively affect communications with communities ( Kraus et al., 2019), and as a result, it will affect social cohesion. A further disadvantage of limited human resources is an absence of competence resulting from a lack of knowledge ( Desouza & Awazu, 2006). SMEs may lack the expertise required to devise and execute successful social cohesion initiatives, frequently lacking access to specialists in diversity, equality, inclusion, or community involvement ( Stockdale et al., 2012). Accordingly, poor service delivery can undermine firm trust and lead to dissatisfaction among consumers ( Singh & Sirdeshmukh, 2000), which weakens social bonds and cohesion.

### 3.2 Limited reach

Typically, SMEs possess a smaller network and constrained visibility, rendering it difficult to engage a larger audience or capture the interest of broader stakeholders. Due to their limited size and diminished impact, SMEs may have difficulties partnering with larger organizations or government entities ( Schweizer, 2013). Indeed, SMEs are often vital for local economic growth, providing jobs and services that benefit communities ( Gherghina et al., 2020). However, SMEs have limited outreach, they may face challenges in creating sufficient job opportunities, resulting in higher unemployment rates and economic inequalities ( Nasr & Rostom, 2013). Consequently, this can worsen social inequality and as a result weaken social cohesion when different groups experience varying levels of economic well-being ( Hulse & Stone, 2007).

Furthermore, SMEs frequently act as a crucial function in indorsing society involvement and social networks. They are commonly more cohesive within local societies than larger firms, engaging in local activities, endorsing local initiatives, and encouraging connections with citizens. However, limited outreach may diminish their capacity to interact with society, undermining social connections and lessening the sense of belonging among its members ( Sen & Cowley, 2013). In addition, SMEs are delivering vital goods and services customized to the desires of local societies. When their reach is limited, some societies, predominantly rural or underserved regions, may be deprived of access to these products and services ( von HØivik & Shankar, 2011). This may result in societal fragmentation, as disparate regions encounter differing access to vital resources, engendering enclaves of deprivation and dissatisfaction ( Sen & Cowley, 2013). It has been claimed that SMEs can serve as catalysts for innovation and social capital, enhancing the vitality and resilience of societies ( Agyapong et al., 2017). Nevertheless, limited reach can hinder innovation and diminish the exchange of ideas and resources within and among societies ( Vrgovic et al., 2012). This can impede the cultivation of social capital, which is vital for trust, collaboration, and collective action ( Bigliardi & Galati, 2016), all of which are important for social cohesion ( Orazani et al., 2023).

Economic volatility and social disparities stemming from the limited influence of SMEs may precipitate political and social unrest ( Brown et al., 2020). That is, when people feel economically marginalized and socially disconnected, they may become more susceptible to extremist ideologies or social unrest ( Jasko et al., 2017; Silver, 1994). This may further undermine social cohesiveness and result in a fractured society ( Jenson, 1998). Furthermore, cultural preservation influences social cohesion ( Jeannotte, 2003), while SMEs repeatedly contribute to the safeguarding and promotion of local customs and traditions ( Yang et al., 2018). However, limited outreach may hinder their capacity to preserve cultural traditions, resulting in the erosion of cultural identity and legacy. This may undermine the social fabric and diminish the feeling of collective identity and purpose within societies ( Ghose & Ali, 2023; Shepherd, 2019).

### 3.3 Reconciling varied interests

In smaller organizations, the probability of internal conflicts or divergent ideas among members is increased, obstructing progress in social cohesion initiatives. Society organizations may encounter difficulties achieving consensus on a delicate social topic, resulting in internal discord ( Caputo et al., 2018; Dolz et al., 2019). Reconciling diverse interests in SMEs poses a considerable challenge to social cohesion. SMEs repeatedly function within varied economic, social, and cultural contexts where stakeholders—owners, employees, customers, suppliers, and local societies ( Sen & Cowley, 2013), which in turn, exhibit conflicting priorities ( Post et al., 2009).

When SMEs neglect to reconcile these conflicting interests, it may result in various repercussions. Firstly, economic inequality exacerbates the disparities among business proprietors, employees, and communities ( Bapuji et al., 2020; Fassin, 2008). Secondly, social fragmentation is marked by a lack of trust among groups, labor strikes, or demonstrations ( Schulman et al., 1999). Third, political polarization juxtaposes business leaders against workers and urban SMEs against their rural counterparts ( Cecora, 2000). Fourth, the disintegration of common values transpires when profit interests supersede societal welfare ( Melé, 2012).

### 3.4 Sustainability

SMEs may encounter difficulties in maintaining long-term social cohesion programs owing to variable resources, volunteer fatigue, or shifts in leadership ( Blackburn & Ram, 2006; Martinez & Aldrich, 2011). Consequently, a smaller organization may initiate a successful program to foster social cohesion but have challenges sustaining it, owing to inadequate recurring financing or volunteer assistance ( Vargas-Hernández, 2023). The sustainability of SMEs is widely recognized as a barrier to social cohesion, mainly because of their challenges in balancing economic viability with environmental and social responsibilities ( Lawrence et al., 2006; Prasanna et al., 2019).

Further, sustainability in SMEs can pose challenges to social cohesion for several reasons, such as Economic limitations restricting social investments, as most SMEs function with little profit margins, hindering their ability to engage in sustainable practices ( Kaousar Nassr & Wehinger, 2016; Santos, 2011). In addition, disparate access to resources for sustainability as Larger enterprises have more significant resources to implement green technology and ethical practices. However, SMEs may be constrained by insufficient finance, knowledge, or governmental assistance ( Bakhtiari et al., 2020; Peter et al., 2018). Moreover, job instability, concerns over labor rights, and the transition to sustainable models may necessitate restructuring, resulting in job losses or precarious employment circumstances ( Carbery & Garavan, 2005). Likewise, supply chain pressures and local societal impact. Most SMEs rely on global supply networks and encounter pressure to comply with sustainability norms, thereby elevating prices ( Huq & Stevenson, 2020; Zaridis et al., 2021). As a result, transferring these expenses to consumers or employees (via reduced salaries) may exacerbate economic inequalities and undermine society cohesion ( Kaymak & Poschke, 2016; Stantcheva, 2022). Besides, insufficient stakeholder involvement and trust deficiencies as SMEs that neglect to include workers, customers, and local societies in sustainability initiatives jeopardize the support of essential stakeholders ( Eikelenboom & Long, 2023). Therefore, skepticism increases without transparent and inclusive policies, eroding social trust and impeding collaborative efforts toward sustainability ( Tsang et al., 2009). Finally, policy and regulatory issues, together with inconsistent or too rigorous rules, can encumber SMEs, compelling them towards non-compliance or informality, so undermining social and environmental accountability ( Favourate, 2021; John, 2024; Mallett et al., 2019; Nieuwenhuizen, 2019).

### 3.5 Scalability

Although SMEs may exhibit innovation, expanding successful efforts to a broader audience or geographic region might be challenging, owing to resource limitations and restricted capabilities ( Gamage et al., 2020; Thorpe et al., 2005). A thriving society program may be beneficial in one neighborhood but not easily repeatable in other areas, owing to variations in local context or resource availability ( Forrest & Wiek, 2014). Scalability poses a significant challenge for SMEs in promoting social cohesion, as their limited reach, resources, and institutional support restrict their ability to achieve large-scale transformation ( Islam, 2022; Osano, 2023). SMEs can overcome these obstacles by leveraging partnerships, adopting flexible models, utilizing technology, and enhancing their influence ( Coleman et al., 2016). While they may never match the scale of large organizations, their localized strength and creativity are crucial for building cohesive societies—one society at a time ( Davenport & Bibby, 1999; Purnamasari, 2024). Figure 2 illustrates the aforementioned challenges that SMEs must overcome to enhance social cohesion.

**Figure 2.**
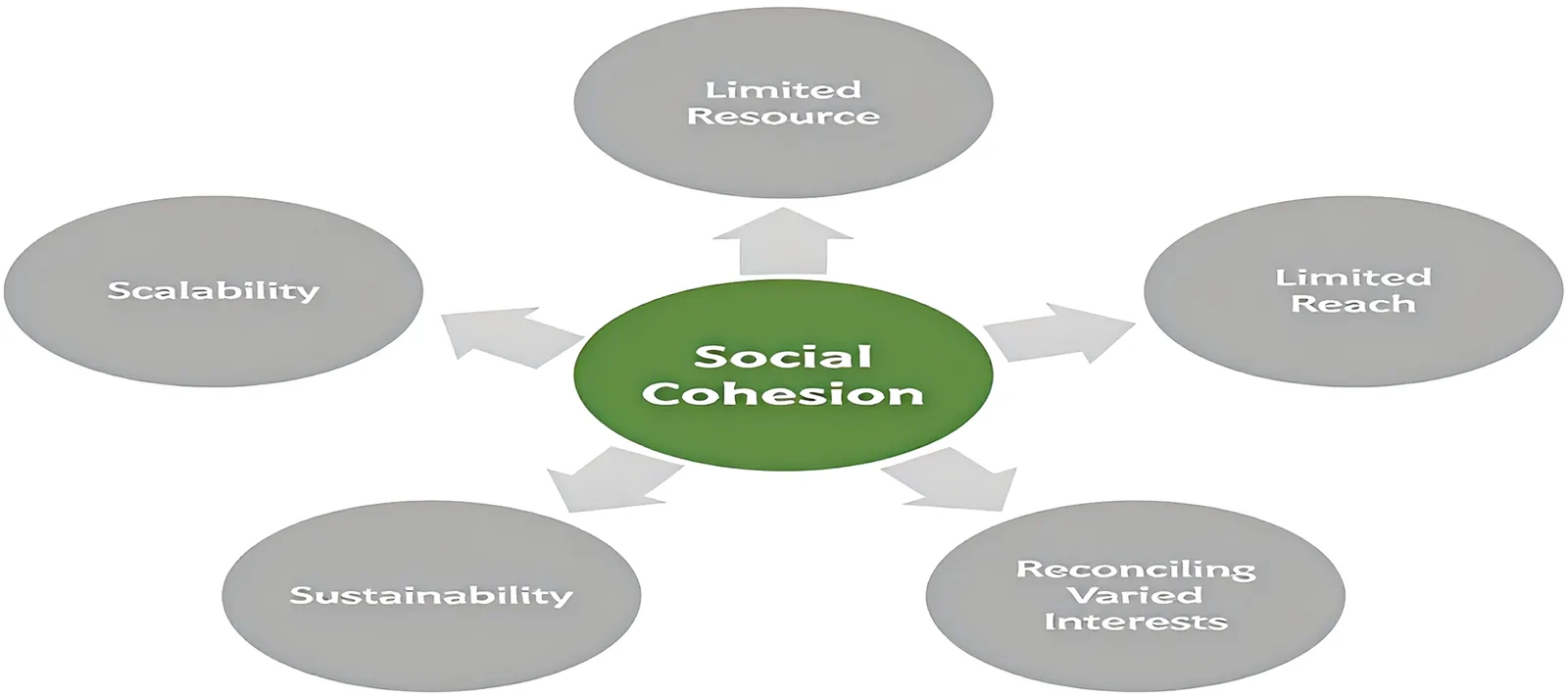
SMEs’ Five challenges that hinder social cohesion.

## 4. Discussion

The current paper is a preliminary attempt to explore and deliberate comprehensively, on the one hand, opportunities that might be beneficial for SMEs in fostering social cohesion. On the other hand, SMEs face challenges that might hinder their efforts to foster social cohesion. This comprehensive view has the potential to provide a better understanding of the ambiguity surrounding this topic, which in turn helps SMEs boost their efforts in enhancing social cohesion in two ways: First, best exploiting the available opportunities that translate efforts into enhanced level of social cohesion. Second, SMEs must overcome the expected challenges that might hinder their efforts to support social cohesion.

Basically, our article mainly reviews major previous work from management and economic, as well as sociological perspectives. Then, we tried to make a bridge between them to represent these opportunities and challenges that SMEs should take into account in their efforts to enhance social cohesion. Primarily, there are five factors determined as opportunities that SMEs should consider to foster social cohesion: Innovation and creativity, flexibility and agility, robust community connections, localized impact, and customized engagement. In contrast, five significant challenges have been identified that SMEs should overcome in their attempts to foster social cohesion: Limited resources, limited reach, reconciling varied interests, sustainability, and scalability. Based on an in-depth and extensive literature review, the opportunities and challenges SMEs might face in their endeavor to enhance social cohesion are represented in Figure 3.

**Figure 3.**
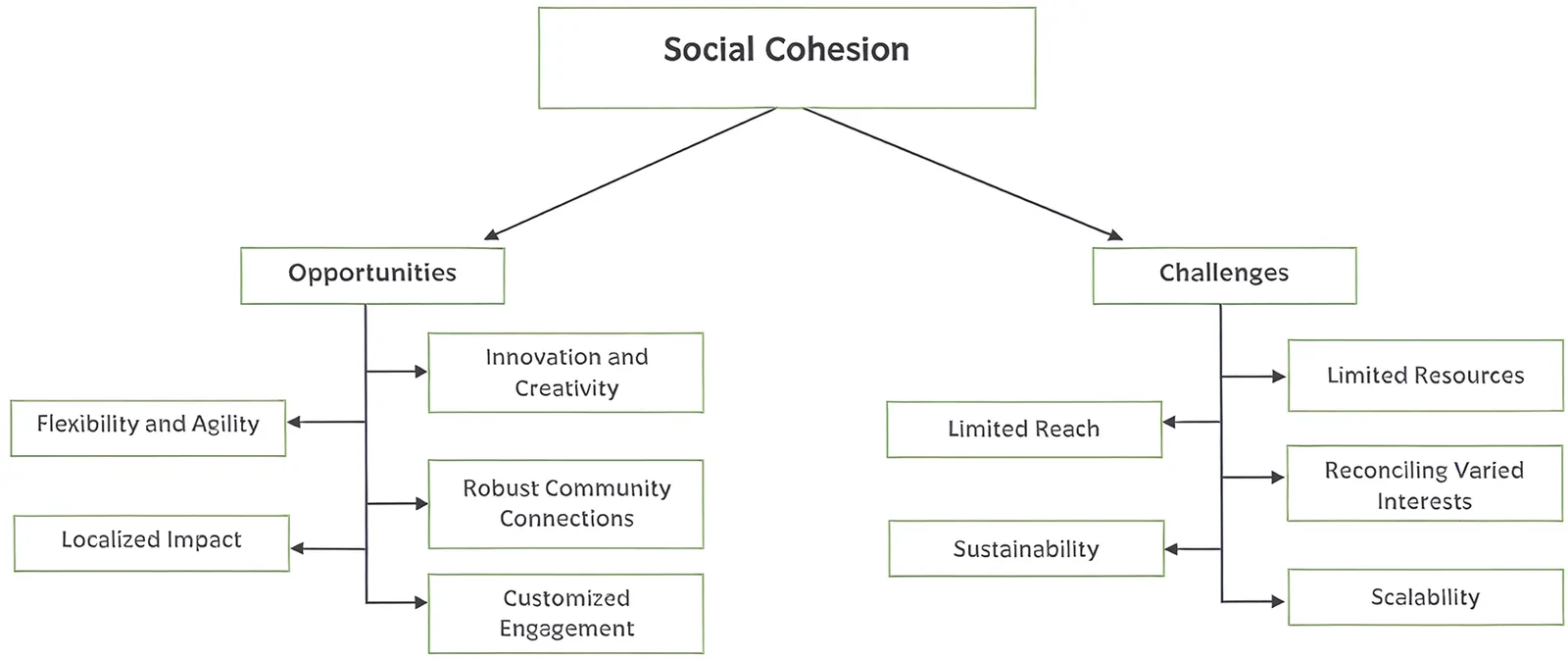
A summary of opportunities and challenges for SMEs toward social cohesion.

In summary, the conceptual model of the current research, which presents propositions that could have positive and negative influences on the role of SMEs in social cohesion, is shown in Figure 4 below.

**Figure 4.**
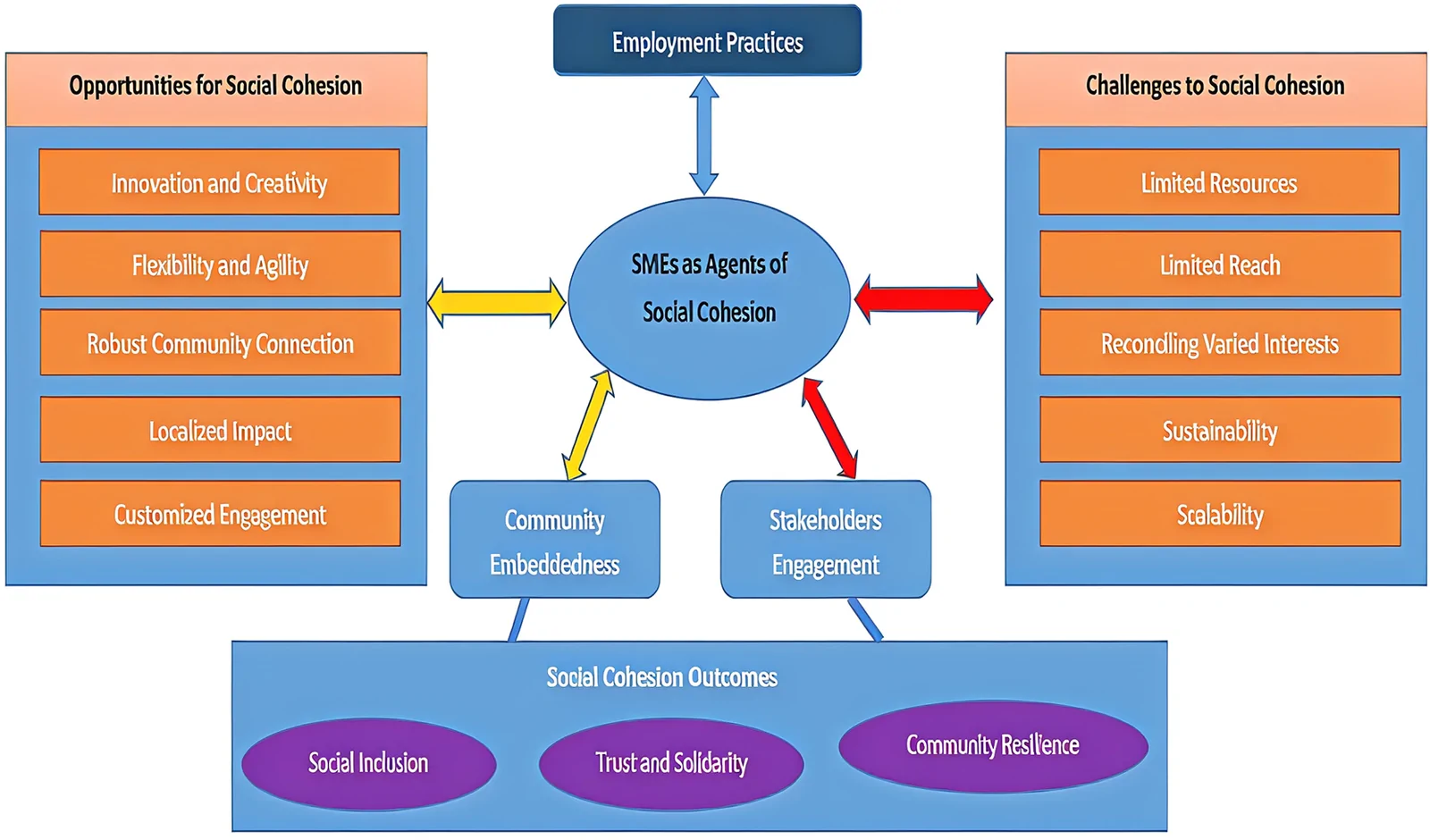
Conceptual framework.

## 5. Directions for future research

The current study considers a type of qualitative research that profoundly depends on discussion and interpretation rather than examinations using a quantitative approach that requires data and generates results through several statistical tests. Consequently, future research might focus on determining which of the aforementioned opportunities and challenges significantly impact fostering or hindering social cohesion. Likewise, a deep systematic literature review is needed for better evidence-based research among different sectors and contexts.

## Data Availability

No data are associated with this article. This study is conceptual in nature and does not involve the collection, analysis, or generation of empirical data. Therefore, no data are available.
