# SMEs as Agents of Social Cohesion: Opportunities, Challenges, and Conceptual Framework

**DOI:** 10.12688/f1000research.183208.2

**Published:** 2026-10-10

**Authors:** Malek Al-Edenat, Nayel AL Hawamdeh

**Affiliations:** 1 Business, Tafila Technical University College of Business, At Tafilah, `Amman, Jordan; 2 Business, Tafila Technical University College of Business, At Tafilah, `Amman, Jordan

**Keywords:** Social Cohesion, Community Businesses, Community Development, Community Innovation, Social Enterprise

## Abstract

Small and medium enterprises (SMEs) are widely acknowledged as key contributors to inclusive and sustainable development. However, their role as agents of social cohesion remains conceptually fragmented and insufficiently developed. This paper addresses this gap by proposing an integrated conceptual framework explaining how SMEs foster social cohesion through local embeddedness, employment practices, and stakeholder engagement. Drawing on interdisciplinary perspectives from entrepreneurship, social capital, and corporate social responsibility, the study identifies key opportunities, including innovation and creativity; flexibility and agility; robust community; localized impact; and customized engagement. It also outlines critical constraints and challenges, such as limited resources, limited reach, reconciling varied interests, sustainability, and scalability. By synthesizing these opportunities and challenges, the paper advances existing literature and offers a coherent analytical model supported by research propositions for future empirical studies. The study provides valuable insights for policymakers and practitioners aiming to position SMEs as effective drivers of social cohesion, particularly within emerging and resource-constrained economies.

## 1. Introduction

In a progressively linked world, social cohesiveness within businesses has become crucial for promoting cooperation and creativity, especially in small and medium enterprises (SMEs). These businesses frequently encounter distinct factors that affect their ability to foster a unified culture, such as resource constraints, employee demographics, and differing stakeholder expectations ( Turner, 2001). As SMEs confront these difficulties, they simultaneously have unprecedented opportunities to utilize their agility and cohesive settings to strengthen social connections and trust ( Lukito-Budi et al., 2023). The interaction between these opportunities and challenges shapes the internal structure of these organizations and has wider ramifications for their sustainability and community impact ( Lee, 2009).

Social cohesion refers to the connections that integrate individuals within a community, fostering mutual respect, trust, and a collective sense of belonging ( Delhey & Dragolov, 2016). Additionally, social cohesion is vital for enhancing collaboration and innovation within SMEs. Furthermore, employees’ connections with each other and their alignment with the organizational mission positively correlate with increased engagement and productivity levels ( Oketch, 2004; Rumasukun & Noch, 2023). Social cohesion can mitigate conflicts and improve communication, thus creating a positive work environment essential for growth and development ( Aall & Crocker, 2019). Therefore, the interaction between social cohesion and organizational success highlights the importance of SMEs prioritizing the cultivation of inclusive and supportive social networks to navigate their unique challenges ( Loukopoulos et al., 2024). For instance, the Danish model shows that higher social solidarity is linked to economic stability, suggesting that cohesive organizations can effectively address external challenges. The association between social cohesion and organizational success emphasizes SMEs’ need to develop inclusive and supportive social networks to tackle their specific difficulties ( Rumasukun & Noch, 2023).

From a different point of view, social cohesion is characterized by a collective sense of belonging within a group, the establishment of bonds and connectedness among community members, and the facilitation of positive social interactions ( Bottoni, 2018). Groups exhibit cohesion when group-level conditions foster positive attitudes and behaviors among members, and when interpersonal interactions sustain these conditions ( Friedkin, 2004). It has been claimed that responsible actions enhance a company’s reputation. Corporate contributions to society are advantageous; hence, fostering social cohesiveness is in the company’s best interest ( Koonce, 2011).

### 1.1 Conceptualizing social cohesion

In this paper, social cohesion is described as the level of confidence, belonging, association, involvement, and commonality in the communities where SMEs are active. The main goal is not internal employee cohesiveness but rather the organizational structure that allows SMEs to contribute to community cohesion in general ( DiBella et al., 2023). Consequently, this paper adopts community-level social cohesion as the main outcome. That is, employee cohesion improves SME functioning ( Drummond & Stone, 2007), which in turn contributes to community social cohesion ( Purnamasari, 2024).

## 2. Opportunities for enhancing social cohesion

### 2.1 Innovation and creativity

SME innovation and creativity have long been seen as distinguishing traits, allowing companies to adapt quickly to changing market conditions while creating economic and social value. Innovation helps SMEs improve organizational performance, social cohesion, and resource mobilization by encouraging collaborative problem-solving and community engagement ( Laforet, 2011). Research reveals that innovative partnerships that foster trust, reciprocity, and collective participation amongst people from different cultural and socioeconomic backgrounds are most effective ( Deakin & Allwinkle, 2007). In the Danish innovation paradigm, collaborative labor-market practices and relatively high income equality provide institutional circumstances that sustain social cohesiveness through shared goals and mutual support ( Lundvall, 2007). Nevertheless, Vieira et al. (2020) propose that creative tourism collaborations boost local identity and community bonds by encouraging cultural and tourist stakeholders to collaborate. These studies suggest that innovation goes beyond technological or commercial outcomes to include social processes that may strengthen social cohesion ( Zahoor et al., 2020).

Innovation is critical to organizational success, yet its effects on social cohesiveness are understudied ( Minhas & Sindakis, 2022). While most studies investigate innovation as a source of competitive advantage or organizational success ( Chatzoglou & Chatzoudes, 2018), few studies investigate how collaborative innovation influences societal consequences ( Olsson et al., 2020). Innovation in SMEs is often fueled by strong links, employee collaboration, and community engagement, rather than traditional research and development ( Annamalah et al., 2025). In this way, the social mechanisms of trust, inclusion, and community participation in innovation are conceptually underdeveloped.

SMEs need employee engagement and teamwork to convert innovation into societal value. The growth of telecommuting and virtual teams raises questions about the pros and cons of collaborative innovation ( Eng et al., 2025). In particular, virtual teams allow SMEs to leverage diverse knowledge, skills, and experiences beyond geographic boundaries, promoting creativity, problem-solving, and organizational learning ( Tang et al., 2020). However, such cooperation is effective only if organizational factors promote trust, communication, team resilience, and diversity ( Gucciardi et al., 2018). These relational skills build trust and cooperation and improve organizational effectiveness and social cohesion ( Lu & Fan, 2017). According to Minhas & Sindakis (2022), leadership can coordinate distributed teams by allocating responsibilities, fostering involvement, and promoting mutual understanding across distances.

Taken together, the existing literature reveals that innovation and employee co-operation are mutually supportive, complementary processes. Innovation is the impetus for collaboration, and a successful collaboration is the relational framework for ideation, sharing, and implementation. However, most research has focused on these qualities separately and rarely described how their interaction facilitates social cohesiveness at the community level. To overcome this conceptual fragmentation, this study combines innovation and collaborative work practices within employment practices, proposing that inclusive and collaborative workplaces are one of the main ways SMEs can promote social cohesion.

### 2.2 Flexibility and agility

SMEs are generally seen as having flexibility and agility as their differentiating factors from larger firms. Firms with less hierarchical structures and closer relationships with their employees and customers can adapt more quickly to the changing economic, social, and community conditions ( Koçyiğit & Akkaya, 2020). This responsiveness enables SMEs to address emerging societal issues through timely interventions, adaptive decision-making, and regionally tailored activities ( Nyamboga, 2026). But evidence suggests that not all SMEs are flexible ( Neirotti et al., 2017). Instead, leadership, strategic direction, organizational culture, and resource mobilization determine their performance ( Chan et al., 2004). Thus, the agility of SMEs is dependent on internal skills and external situations ( Liu & Yang, 2020).

Research consistently demonstrates that SMEs have organizational characteristics that enable agility. Their simplified structures and low hierarchy allow them to make decisions faster and reduce administrative delays ( Zaridis et al., 2021). Employees often hold several roles, promoting cross-functional collaboration and organizational growth. Strong customer relationships help SMEs to adapt their products, services, and social initiatives to the evolving needs of the community and market ( Konsti-Laakso et al., 2012). Rigid bureaucratic procedures do not hinder SMEs and can change operational processes, reorganize supply chains, and better deal with unexpected interruptions ( Nakandala et al., 2025). Entrepreneurial leadership enables quick strategic changes in response to changing environmental conditions, promoting experimentation and innovation ( Shaw, 2014). These features make SMEs more resilient to economic crises and other external shocks ( Falkner & Hiebl, 2015; Klein & Todesco, 2021).

There is a broad consensus on the adaptive benefits of SMEs ( Quansah et al., 2022); however, the concepts of flexibility and agility have been addressed mainly in terms of organizational resilience, innovation, and competitive performance. Little attention has been paid to understanding how these qualities affect social outcomes. Rapid adaptability helps SMEs preserve employment, community links, and social needs in the face of disruptions, but research rarely explains how ( Klein & Todesco, 2021). Organizational flexibility can increase social cohesion, as SMEs can maintain local employment, stakeholder relationships, and community-oriented projects, whilst larger organizations are limited by more complex organizational structures ( Jennissen et al., 2023). Flexibility is not always a good thing ( Golden & Powell, 2000). Agile organizational structures allow for emergency responses to changing conditions but may be limited in impact by a lack of financial, managerial, technical, and institutional support ( Janssen & van der Voort, 2020). Hence, the impact of organizational flexibility on social cohesion may be contingent on SMEs’ adaptive skills and their economic and institutional setting. Employment Practices, therefore, comprise flexibility and agility, where adaptive organizational arrangements, collaborative working environments, and responsive leadership help to strengthen SMEs’ ability to contribute to social cohesion at the community level ( Holbeche, 2018).

### 2.3 Robust community connections

A common thread running through the research is that SMEs can develop deep individual relationships with the neighborhoods in which they operate. Such interactions can provide opportunities for establishing confidence, a sense of belonging and inclusion, and allow SMEs to tap into community stakeholders, social networks, and local expertise. ( Spence & Schmidpeter, 2003). But we should not jump to the conclusion that these linkages would necessarily translate into increased social cohesiveness. Instead, community linkages are most valuable when SMEs are well embedded in local social and economic systems. This allows them to identify community needs and participate proactively in finding solutions. This distinction is important because most of the existing research on SMEs focuses on their economic importance as employment generators and contributors to local development ( Muriithi, 2017; Robu, 2013; Surya et al., 2021).

The study results show that geographical and institutional factors affect the quality of the relations between SMEs and their communities. In rural areas, where professional, social and familial links are more likely to be intertwined than in larger and more dispersed companies, SMEs and their owner-managers may become more entrenched ( Child et al., 2022; Korsgaard et al., 2015; Williamson et al., 2006). If SMEs are more entrenched in the local economy, culture, society and environment, they may be more incentivized to help their communities grow. Rural SMEs can play a role in local development by creating jobs, taking part in community-based activities, and innovating to respond to locally specific needs. ( Cravo et al., 2012; Lang et al., 2014). Meanwhile, studies show that rural areas have unique physical, cultural, and historical resources that SMEs may use to create social and economic projects relevant to their communities ( Korsgaard et al., 2015; Manzoor et al., 2021). And this type of community embeddedness may be reinforced even more by family ownership. In the case of the owner-manager of SMEs where the whole family lives in the same town, a person’s professional name can become blurred with the personal name. Maintaining good relations with neighborhood people and other members of the community might, therefore, have implications beyond the bottom line ( Banki & Ismail, 2015; Larsson et al., 2003). Recent research suggests that being embedded can give SMEs in rural areas an added advantage, with ongoing connections with residents helping them gain a better awareness of local conditions ( Kaban et al., 2026). SMEs that are aware of their communities may be better positioned to exploit opportunities that are unique to their locality and exploit resources that are specific to their culture, history and geography ( Beckmann et al., 2023; Korsgaard & Tanvig, 2015).

But the evidence is not to be taken as showing that proximity necessarily produces beneficial social effects. Community embeddedness is likely to affect trust and collective involvement in different ways depending on community characteristics, the strength of relationships between SMEs and local stakeholders, and SMEs’ capacity to respond to local demands. The relationship between embeddedness and social cohesiveness may depend on context, with the strongest evidence coming from SMEs owned by families or located in rural areas. This finding reveals a major lacuna in the extant literature: while local embeddedness of SMEs is acknowledged, very little attention has been paid to how this embeddedness is reflected in social cohesiveness at the community level.

Taken together, linkages between SMEs and their communities are more than spatial proximity. This includes the ability to mobilize community-specific resources, shared social contexts, local knowledge, and long-standing relationships. These characteristics can potentially enable SMEs to promote a greater sense of community identification, trust, inclusion and engagement. The research regards strong community ties as an important part of the wider Community Embeddedness mechanism that SMEs can potentially use to enhance societal cohesiveness. This way of thinking also explains that the effectiveness of this mechanism will depend on a range of environmental factors, including but not limited to location, ownership composition, and the strength of ties between SMEs and local stakeholders.

### 2.4 Localized impact

Second, SMEs can help bring communities together by tailoring their products and services to local needs and customizing their hiring policies, community service programs, and other policies to the peculiarities of each location they serve. SMEs with a local focus are better able to respond to the economic, social, cultural and environmental conditions of particular communities than are standardized interventions designed for large markets or widely dispersed populations. Studies on regional development and local economic systems suggest that place-specific competences develop and interactions between local actors are promoted through the geographical concentration of economic activity. ( Harte et al., 2016; Makkonen & Leick, 2020; McCann & Ortega-Argilés, 2016). From a social cohesion perspective, it is essential to note that SMEs are not only physically close to communities but can also convert local knowledge into activities that community members perceive as relevant. This can lead to more meaningful and relevant social cohesion interventions. Localization speeds up brand expansion and makes products more accessible to consumers. Likewise, evidence on variations between urban and rural SMEs suggests that location can create both opportunities and constraints for SME development ( Freeman et al., 2012). Nevertheless, the research shows that the benefits of localization depend on the circumstances. SMEs working on a smaller geographic scale may be better positioned to identify specific community needs based on the particulars of the local market, institutional conditions, and the resources they have available. ( Chhibber & Chadha, 2020).

Therefore, the organizational impact does not apply to all SMEs. Rather, the advantages of localization are more evident when SMEs can adapt their operations to specific regional needs. This matters because community bonds can be enhanced by the capacity to translate local presence into meaningful, inclusive economic and social contributions, rather than simply being physically proximate.

One example of how the activities of SMEs within a region can potentially serve the broader society is job creation. It is well known that SMEs help people find work and boost local economies, especially in marginalized communities. One example is Northern Uganda, where women business owners were able to secure microfinance based on social collateral from their existing social networks, helping conflict-affected communities recover their livelihoods ( Bongomin et al., 2020). These findings show that localized economic activity can promote social cohesiveness by providing opportunities for economically marginalized groups rooted in existing community bonds. Likewise, there is evidence of increased employment and overall economic stability in Jordan for SMEs, but further research is needed to establish the specific impact on social cohesiveness ( Wolfe, 2016). These findings consequently strengthen a possible rather than deterministic relationship between localized SME activity and social cohesion.

Together, SMEs and other community members can potentially create a more localized impact. Community priorities include education, infrastructure, and livelihood development. Local actors can pool their resources and skills to solve these problems through partnerships between NGOs, grassroots organizations, and SMEs. Such collaboration can foster opportunities to build confidence and work together, especially when community members help identify problems and develop solutions. Participation in corporate social responsibility initiatives has been shown to benefit SME employees through improved morale and stronger relationships with the communities in which they live, as employee engagement in CSR activities undertaken by SMEs increases morale and establishes links between the firms and the community ( Smith et al., 2022).

However, there should be a conceptual difference between these interactions. In this study, NGOs and grassroots groups are not seen as potential alternative focal organizations, but rather as external stakeholders with whom SMEs may engage. This differentiation keeps SMEs as the key unit of analysis, while inter-organizational interactions are important in producing localized social effects.

The localized nature of SME activity may also affect cultural and social factors. SMEs that are active in a given community can help keep local cultural resources alive and well, while also providing opportunities for people from all walks of life to meet and engage. Research has found that community-based spaces may foster cross-social engagement, while local economic development may foster cultural exchange and place-based identity ( Komorowski et al., 2023). Similarly, it has been argued that this cultural exchange strengthens one’s sense of identity and reduces social conflict. SMEs play a vital role in rebuilding social networks in war-ravaged areas ( Joseph & Van Buren, 2022). Localized SMEs can replace fragmented systems with collaborative ones. For example, women’s micro-enterprises in these areas rely on social cohesiveness to secure loans and sustain their businesses. As per Djip (2014), these SMEs offer material and emotional stability for the healing of fractured communities. Last but not least, SMEs often focus on environmental efforts, such as buying supplies locally, to reduce carbon footprints. “Farm-to-table” businesses buy their food directly from the farm, linking economic growth with environmental protection, and supporting organic farming and community self-sufficiency. These initiatives create a sense of collective environmental responsibility and bring communities together around common values ( Agan et al., 2013).

Overall, the research suggests that localized impact is a way for SMEs to leverage their proximity to particular communities to make social, cultural, economic and environmental contributions that matter to those communities. The relationships, trust and local knowledge that SMEs develop through community embeddedness are important for strong community connections. Still, the translation of those relationships and knowledge into context-specific actions and outcomes is the focus of localized impact. This difference sheds light on the reciprocal nature of the two possibilities. SMEs that are well-connected within their communities are likely to be better placed to understand and authentically respond to local objectives. The localized impact they generate is a direct result of how they adapt their operations to meet those needs. This study therefore sees localized impact as an important opportunity for SMEs to contribute to social cohesion through the larger Community Embeddedness mechanism. This is particularly the case where there is a good match between local knowledge, stakeholder participation and organizational resources.

### 2.5 Customized engagement

Personalized interaction is another opportunity for SMEs to engage their customers, employees, and community members. Because they tend to operate in more limited markets and have stronger personal relationships than large organizations, SMEs may have more capacity to adjust their interactions to the requirements of various stakeholder groups. Such involvement helps build trust, engagement, and a sense of ownership when stakeholders feel their opinions are heard and integrated into the business operations. One way to do this is through a mentorship program that pairs people from different backgrounds, which SMEs, advocacy groups, and community members could develop together. ( Iyelolu et al., 2024; Wee & Chua, 2013). These procedures propose that customized engagement can widen beyond customer relationship management to serve as an instrument for enabling interaction and participation across social groups.

The proliferation of digital technologies has enhanced SMEs’ ability to customize stakeholder interaction. Data-driven tools, artificial intelligence (AI), and digitization can help SMEs better understand consumer needs, customize their communications, and provide more personalized interactions. The literature suggests that the social benefit of personalization is contingent upon the integration of these technologies in organizational interactions. Customization can make things more relevant and easier to access, but technology interactions alone cannot guarantee the development of trust, inclusion, or community engagement. Therefore, for digital engagement to become a possible driver of social cohesiveness, SMEs need to use technology to elevate authentic human connection, not only automate it ( Abrokwah-Larbi & Awuku-Larbi, 2024; Kedi et al., 2024a; Telukdarie et al., 2022).

Social media has provided new avenues for encouraging participation for SMEs. User-generated material, customer stories, interactive polls, and platform-tailored messaging could offer users a chance to participate and share their experiences ( Ohara et al., 2024; Qalati et al., 2021, 2022). Similarly, SMEs may reach out to a wider audience by tailoring their material to different platforms and responding promptly to online comments and discussions, thereby establishing credibility and openness ( Kedi et al., 2024b; Kraus et al., 2019; Huang & Liu, 2021). What these practices do to turn the stakeholders from passive receivers of organizational information into active participants in joint discussions is more important than the technology itself from the viewpoint of social cohesion. Such interactive communication can strengthen community stories and connections between people ( Belair-Gagnon et al., 2019).

In a similar vein, other studies have shown that SMEs can benefit from co-creation by including stakeholders in the innovation process from the very beginning. Stakeholders can have a say in organizational decisions through feedback systems like surveys, polls, and other types of direct consultation, and social customer relationship management can help incorporate customer knowledge into product and service development ( Hassan et al., 2019; Bodó et al., 2019). Fernandes & Remelhe (2016): Customers’ perceptions of their ideas and experiences being recognized and integrated into new products or services through co-creation may also increase stakeholders’ emotional investment in organizations. In a broader sense, SMEs may be able to build relationships with their communities by integrating data from various communication channels to provide stakeholders with more consistent and tailored experiences ( Hayes & Kelliher, 2025).

A critical tension between technology efficiency and relational authenticity is highlighted in the literature as an issue with customized interaction. SMEs may more efficiently recognize positive or negative reactions with the help of AI-supported communication, which improves responsiveness and makes stakeholder sentiment analysis easier ( La Sala et al., 2024; Egger et al., 2019). Localized content in which SMEs modify automated material to embody local subtleties, guaranteeing inclusion ( Raji et al., 2024). Consequently, this equilibrium guarantees that technology augments, rather than supplants, the human element, which is essential for social cohesiveness.

The literature suggests that through personalization, participation, co-creation, and digitally connected communication, customized engagement can help SMEs to build stakeholder connections. The results show that customized participation does not necessarily result in social cohesion. The possible effect seems to depend on stakeholder participation, authenticity, the use of digital technology, and the SMEs’ ability to balance automation and human reactivity. This distinction is important because most research has examined personalization, social media engagement, customer relationship management and co-creation in relation to customer satisfaction, innovation and firm performance, but not at the community level of social cohesion.

The study says customized engagement is an option under stakeholder engagement. Community Embeddedness assesses the level of integration of SMEs in local social and economic environments, while stakeholder engagement means involving stakeholders in organizational and community activities. Customized engagement can lead to participative and reciprocal stakeholder interactions. SMEs can contribute to social cohesion by creating a space for different stakeholders to interact, collaborate, co-create, and develop shared understandings. These results are contingent on SMEs’ organizational and contextual factors.

In conclusion, SMEs have the potential to enhance social cohesion within the communities they operate in through five opportunities: enhanced innovation and creativity through collaboration and teamwork among employees, flexibility and agility, robust community connections, localized impact, and customized engagement. Figure 1 illustrates the above-mentioned opportunities for SMEs in their endeavor to enhance social cohesion.

**Figure 1.**
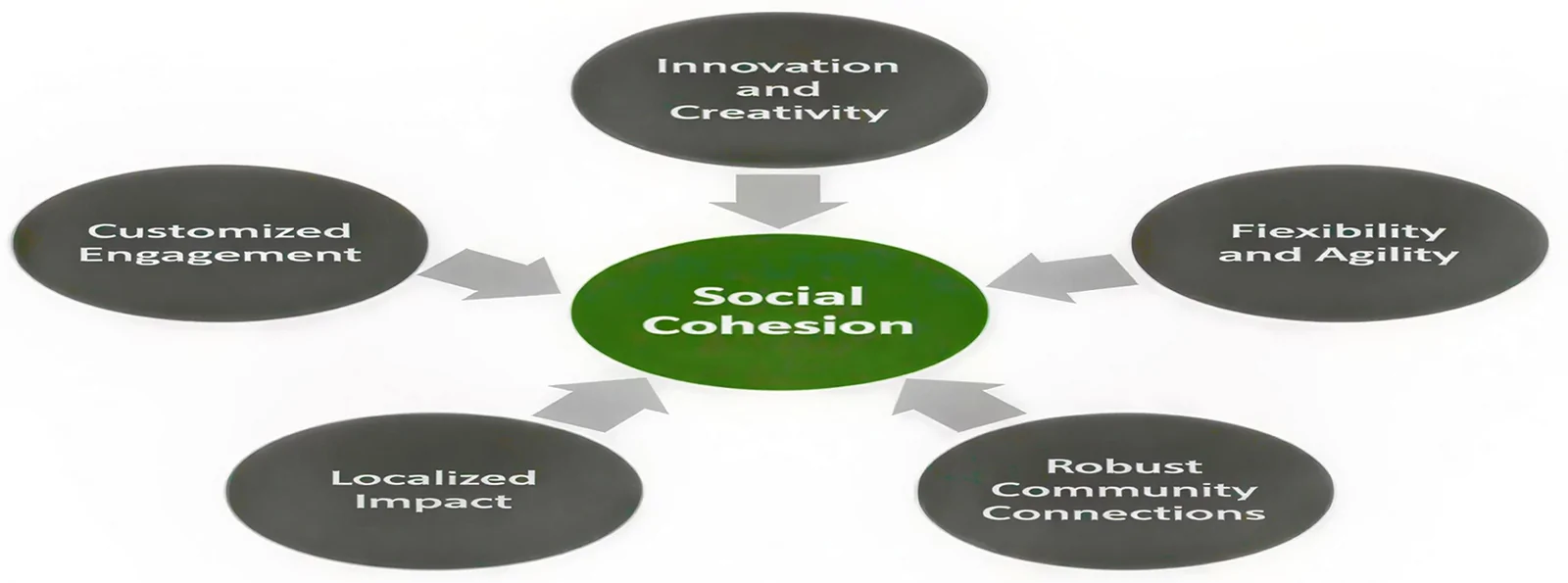
SMEs’ Five opportunities to enhance social cohesion.

## 3. SMEs and Social Cohesion: Three Mechanisms

In light of what has been said thus far, it is clear that the five opportunities highlighted in this analysis are not independent variables but rather complementary aspects that, when taken as a whole, enhance SMEs’ ability to promote social cohesiveness ( DiBella et al., 2023). Employment Practices, Community Embeddedness, and Stakeholder Engagement are three overarching organizational mechanisms that SMEs use to impact social cohesion ( Rumasukun & Noch, 2023). Each opportunity contributes in its own way, but they inevitably converge ( Rumasukun & Noch, 2023). To better understand how SMEs convert organizational strengths into societal consequences, these higher-order mechanisms provide a more concise and theoretically consistent picture. By focusing on their underlying functions and contributions to community-level social cohesion, the suggested framework aggregates these opportunities rather than treating innovation, flexibility, community connections, localized effect, and customized involvement as independent drivers. In conclusion, SMEs have the potential to enhance social cohesion within the communities they operate in through five opportunities: enhanced collaboration and teamwork among employees, flexibility and agility, robust community connections, localized impact, customized engagement, and innovation and creativity.

Employment practices that promote inclusive workplaces, trust, and information sharing include options for innovation through employee co-operation, teamwork, organizational flexibility, and agile work arrangements ( Findlay et al., 2025). That is, SMEs have strong links with their community and can influence what happens locally. SMEs understand what people need locally, can create jobs, retain a local identity, and help to develop the community ( Madden et al., 2006). This is captured by community embeddedness. Last but not least, stakeholder engagement reflects SMEs’ ability to build significant relationships with consumers, vendors, community members, and local institutions through tailored engagement, collaborative innovation, and participative decision-making ( Matarazzo et al., 2025). Together, these three processes show how the five opportunities are interdependent pathways that SMEs can take to improve social cohesiveness, community involvement, trust, and inclusion. This conceptual integration is also theoretically grounded in the conceptual framework of Figure 4.

## 4. Challenges facing SMEs in enhancing social cohesion

In this section, we will shed light on the challenges, or in other words, determinants, that could hinder SMEs in their pursuit of achieving the best level of social cohesion. These challenges will be critically discussed to shape understandable and clear perceptions for SMEs about these challenges that may prevent these firms from achieving a high level of social cohesion. In addition, these perceptions may provide scope for drawing a roadmap that may benefit these companies to overcome these challenges.

Enhancing social cohesiveness in SMEs encounters several hurdles stemming from both internal dynamics and external forces. A significant challenge is the intrinsic variety inside organizations, which, although advantageous for creativity, may also result in misunderstandings and disputes among individuals from varied backgrounds ( Yahaya & Nadarajah, 2023). Furthermore, as mentioned above, the Danish model emphasizes that the economic background indicates that although high-income equality and stability promote social cohesion, economic development stagnation may jeopardize these circumstances’ maintenance ( Lundvall, 2007).

### 4.1 Limited resources

Resources limit SMEs’ capacity to maintain social cohesion efforts. SMEs, particularly in developing countries, have limited financial, human, material, and expertise resources compared to larger organizations ( Prasanna et al., 2019). One illustration of the limited capacity of smaller firms is when they are not in a position to fund a major community event that unites different groups ( Blackburn & Ram, 2006). SMEs may struggle to maintain corporate social responsibility (CSR) and community projects that demand ongoing investment, especially when they conflict with operational and financial priorities, according to previous studies ( Jenkins, 2006, 2009; Sweeney, 2007). Similarly, Nicolas (2022) highlights the fact that small and medium-sized enterprises (SMEs) could have trouble allocating funds to projects whose returns don’t materialize for a while. Consequently, it’s not so much a lack of desire to help achieve social goals as a lack of organizational resources to keep people working toward those goals. This limitation may also mean less money for community embeddedness and stakeholder engagement. Employment Practices may be less able to foster inclusive and collaborative workplaces. This explains why SMEs have difficulty translating their social advantages, such as flexibility, local knowledge, and strong ties with other stakeholders, into long-term social benefits in resource-constrained contexts. The literature suggests that SMEs may not be able to leverage the potential of organizational proximity and responsiveness due to their limited resources ( Jenkins, 2006, 2009; Sweeney, 2007).

Another problem is the shortage of human resources. In SMEs, there is usually little scope for activities beyond immediate operational duties, given the small teams and the employees who perform various tasks ( Desouza & Awazu, 2006). Thus, there is a lack of dedicated employees. SMEs tend to have smaller teams and multitasking employees ( Santos, 2011). This means that SMEs’ employees do not have the time or resources to commit to projects that require special skills, such as social media marketing ( Ramsay, 2010). Kraus et al. (2019) report that staffing constraints may lead to a reduction in community outreach, which in turn affects social cohesiveness. Another disadvantage of a lack of human resources is a lack of expertise, leading to incompetence ( Desouza & Awazu, 2006). The problem extends beyond the number of available employees to the availability of specialized knowledge and expertise. SMEs may have limited capacity to employ or develop specialists in areas such as diversity and inclusion, community engagement, digital communication, and other socially oriented functions ( Stockdale et al., 2012). SMEs may have difficulty implementing and creating socially inclusive projects due to a lack of knowledge. This can harm customer service and relations with community members. Poor service performance can erode consumer trust and happiness, and there may be relational and operational implications for poor organizational capacities. The lens of social cohesiveness can be used to interpret how lower trust and inconsistent stakeholder relationships may undermine SMEs’ ability to sustain the collaborative ties that support community participation and collective action ( Singh & Sirdeshmukh, 2000), which weakens social relationships and cohesion.

Without understanding, it can be challenging for SMEs to create and implement socially inclusive projects. This could negatively affect customer service and community relations. Organizational weaknesses: relationships and operations can be impacted. Poor service performance can lower customer trust and satisfaction. Poor trust and uneven stakeholder relationships may impede the maintenance of collaborative links for community engagement and collective action for small and medium-sized enterprises (SMEs). This can be explained in terms of social cohesiveness. All things considered, SMEs may be unable to play their full role in promoting social cohesion due to limited resources, which limit their capacity to engage stakeholders over the long term, sustain community-oriented events, and ensure equitable employment practices. Consequently, the three mechanisms suggested in this study are directly affected by the challenge. Limited resources can limit investment in employees and specialized capabilities, which in turn limits employment practices. Community embeddedness suffers as a result; as local initiatives cannot be sustained. Stakeholder engagement suffers as a result, as there is a lack of time, expertise, and financial resources to maintain meaningful relationships. Therefore, it is crucial to take resource availability into account as a contextual factor that influences the possibility of SMEs’ social contributions leading to long-term social-cohesion results.

### 4.2 Limited reach

SMEs have a local focus, which is great for community involvement, but their size, exposure, and network may limit the scope and variety of their social impacts. Establishing partnerships with larger firms, government agencies, and stakeholders located in different locations can be challenging for SMEs with smaller organizational networks ( Castilla-Polo et al., 2026; Schweizer, 2013). The same local concentration that may allow SMEs to spread their impact outside their immediate environment can also hinder them from doing so, creating an important tension within the SME-social cohesion relationship. Because of this, SMEs may excel at meeting specific needs in their immediate areas, but they may struggle to rally support for larger-scale projects or coordinate their efforts among community members.

Employment and local economic growth are particularly affected by the implications of restricted reach. They play a significant role in the economy and the labor force; SMEs may be limited in the services they can offer and the number of jobs they can create due to their smaller size ( Gherghina et al., 2020). These findings imply that limits in job creation can have ramifications for unemployment and economic inequality ( Nasr & Rostom, 2013). When some groups are consistently left out of local economic activities, as might happen when economic possibilities are not distributed evenly, it can erode feelings of inclusion and engagement, which is crucial from a social-cohesion standpoint ( Hulse & Stone, 2007). That said, there is no evidence in the literature that social inequality is a direct result of SMEs’ restricted reach. Instead, it implies that the impact of SMEs on employment and inclusion is limited by their ability to operate in the market and within their organizations.

Additionally, SMEs may find it challenging to establish and sustain larger social networks due to their limited reach. Previous studies have shown that small and medium-sized enterprises (SMEs) can make a positive impact on their communities through getting involved in grassroots efforts and forming connections with locals. Such involvement can strengthen social bonds and build local social capital. Concurrently, small and medium-sized enterprises (SMEs) with limited network access may miss out on opportunities to collaborate on projects beyond their local areas, share information, or connect with other groups ( Lashitew et al., 2024). On the other hand, Sen & Cowley (2013) highlight the importance of SME-community relationships, while research on SME networks suggests that restricted access to external relationships can constrain knowledge sharing, innovation, and resource exchange. Thus, limited reach may not necessarily undermine SMEs’ local relationships, but it can restrict the breadth and diversity of the networks through which those relationships generate wider social value. SMEs are the backbone of local communities, providing essential goods and services. Their reach may be limited such that some groups, particularly those in rural or underserved areas, may not be able to access these products and services ( von HØivik & Shankar, 2011). The literature likewise recommends that SME networks can support social capital, innovation, and local economic vitality ( Agyapong et al., 2017). However, when firms have limited access to external networks, opportunities for inter-organizational knowledge exchange and resource sharing may be reduced ( Vrgovic et al., 2012). Such constraints may indirectly influence the relational foundations of social cohesion by preventing opportunities for trust-building, cooperation, and collective action across organizational and community boundaries ( Bigliardi & Galati, 2016; Orazani et al., 2023). The takeaway here is not that SMEs with limited reach inevitably undermine social cohesion, but rather that if their networks stay small and disconnected from outside actors, they may be unable to contribute to broader forms of cohesion.

Economic and social exclusion can have far-reaching effects, which is why it’s important to consider the size of SME networks. Research ( Brown et al., 2020; Jasko et al., 2017; Silver, 1994) links economic marginalization and social isolation to social unhappiness and instability. Jenson (1998) correspondingly underlines the importance of inclusion and connectedness in maintaining cohesive societies. Nevertheless, these relationships extend well beyond the direct influence of SMEs, and the reviewed literature does not justify attributing political or social unrest directly to SMEs’ limited reach. Cultural factors further highlight the possible restrictions of SMEs’ limited reach. There is evidence that SMEs can help maintain social cohesiveness by passing on and preserving local traditions, cultural practices, and place-based identities ( Jeannotte, 2003; Yang et al., 2018), and SMEs provide a continuous flow of support to local traditions and practices. Cultural identity and legacy may deteriorate if SMEs are unable to reach out sufficiently to sustain cultural practices. This may lead people to feel less connected to their community and less invested in its future ( Ghose & Ali, 2023; Shepherd, 2019).

Overall, notwithstanding the benefits of a local focus for SMEs, Limited Reach is a major obstacle to their expansion. While SMEs may have deep roots in their communities and specialized knowledge, they may not be able to reach as many people or have as much of an impact due to their smaller networks, narrower focus, and lack of power. As a result, if SMEs are unable to reach a wide variety of external partners, institutions, and communities, Stakeholder Engagement may suffer, and Community Embeddedness may be unable to produce as many positive social consequences. Limitations on the size of the market can affect employment practices when small and medium-sized enterprises (SMEs) are unable to offer as many or as varied job opportunities. Therefore, it is important to consider the depth and breadth of the networks that SMEs rely on when assessing their impact on social cohesion, in addition to the strength of their local relationships.

### 4.3 Reconciling varied interests

A key obstacle to SMEs’ potential contribution to social cohesion is the multiplicity of stakeholder interests within and surrounding them. The goals and expectations of the various stakeholder groups with whom SMEs often deal—owners, workers, consumers, suppliers, and community actors—are not necessarily congruent ( Post et al., 2009; Sen & Cowley, 2013). Such connections can give SMEs access to new information, contacts, and social support. Still, they can also place pressure on existing goals within the firm and make it more difficult to distribute financial and other rewards equitably. So, it’s not enough that there are competing interests; the key is that SMEs can manage these competing interests and make judgments that stakeholders view as fair and inclusive.

Research has shown that conflicts between organizations and groups of stakeholders may arise from disagreements over social responsibilities, the allocation of resources, or divergent values ( Caputo et al., 2018; Dolz et al., 2019). These tensions may be particularly difficult to manage in the SME setting, considering that SMEs tend to be less formally governed and have more concentrated ownership structures than larger organizations. So, owner-managers have a lot of say in big-picture decisions; even workers, customers, suppliers, and community members may have different ideas about the company’s social and financial responsibilities. SMEs have the strength of being able to respond quickly to the needs of stakeholders. A potential weakness is resolving conflicts between stakeholder needs ( Vargo & Seville, 2011).

Unresolved disputes between stakeholders may have implications far beyond the decisions made by particular organizations. When economic interests are prioritized over the interests of a broader group of stakeholders, the distribution of responsibilities and rewards among owners, employees, and communities can create feelings of injustice or exclusion. From a social-cohesion perspective, a major concern is that when stakeholders experience that their interests are continuously ignored, trust and collaboration could be undermined. As is evident in the literature reviewed here, social fragmentation, labor disputes, and political polarization are not necessarily the consequences of stakeholder conflict. These wider implications are better seen as potential outcomes under some conditions than as direct consequences of disagreements between SMEs ( Bapuji et al., 2020; Fassin, 2008). The situation is further complicated by a lack of consensus between stakeholders regarding the principles and objectives they bring to bear when making decisions that affect SMEs. While stakeholders may not agree on the allocation of resources or social issues, they may agree that community development is important. Consider the challenges small- and medium-sized enterprises (SMEs) might encounter when attempting to reconcile investments in activities that yield short-term financial returns with those that deliver long-term societal benefits. It can be especially hard for organizations to balance their economic and social aims when resources are scarce, and this is where the tensions arise. Melé (2012) emphasizes the importance of integrating ethical and social considerations into organizational decision-making, suggesting that an exclusive emphasis on economic interests may undermine broader stakeholder responsibilities.

Diversity of stakeholder interests should not be seen as a constraint. Different hobbies can expose SMEs to a variety of knowledge, experience, and community views. When SMEs develop effective discussion and engagement mechanisms, stakeholder differences can be converted into collaborative problem-solving. This insight links this difficulty to customized engagement. SMEs can use meaningful engagement with interested parties to uncover conflicting expectations, negotiate priorities and develop solutions from multiple perspectives. Conversely, bad or symbolic engagement practices could increase stakeholders’ distrust and limit SME’s contribution to social cohesion ( de Oliveira Santos Jhunior et al., 2026).

The main challenge of the proposed conceptual framework is to balance different interests that can limit or improve the Stakeholder Engagement process. Diversity can help to learn, collaborate, and arrive at mutually acceptable solutions through inclusive involvement where stakeholder interests are recognized and negotiated. Unresolved interests can lead to exclusion or unfairness, which can erode trust and collaboration. Thus, SMEs contribute to social cohesion by their ability to manage and resolve conflicts between stakeholders in ways that maintain participation, fairness and constructive relationships.

### 4.4 Sustainability

The literature shows that SMEs are not able to maintain social and community-oriented activities. Social initiatives require financial, human and management resources to be maintained (as opposed to initial implementation). Resource limitations, volunteer or staff fatigue, and leadership turnover in the post-implementation phase may limit community-oriented projects of SMEs ( Blackburn & Ram, 2006; Martinez & Aldrich, 2011). Vargas-Hernández (2023) stated that even successful social initiatives can be hard to maintain, highlighting the difference between starting and maintaining a social cohesion contribution. When social initiatives conflict with operational and financial concerns, SMEs may struggle. The conflict at the heart of this challenge is between broader social and environmental responsibilities and the need to remain economically viable. The ability of SMEs to absorb the costs of sustainability-related expenditures is typically limited due to their comparatively constrained financial resources. As a result, SMEs with thin profit margins may face short-term financial constraints when adopting environmentally and socially responsible practices ( Kaousar Nassr & Wehinger, 2016; Santos, 2011). Equally, organizations may struggle to balance economic goals with environmental and social responsibilities. These pressures suggest that SMEs’ potential contribution to social cohesion cannot be considered independently of their capacity to remain economically viable. A socially oriented initiative that cannot be sustained financially may have limited long-term effects ( Lawrence et al., 2006; Purnamasari, 2024).

Disparities in resources and competencies between larger organizations and SMEs further define the sustainability dilemma. When it comes to adopting green technology and implementing formal ethical and sustainable policies, larger firms may have more financial, technological, and managerial capabilities than SMEs. SMEs may also encounter obstacles relating to knowledge, funding, technical expertise, and institutional support ( Bakhtiari et al., 2020; Peter et al., 2018; Kulkarni et al., 2024). Such differences should not be taken as evidence that SMEs are inherently less sustainable. Rather, they indicate that the organizational resources needed to implement and maintain sustainability practices are unevenly distributed. This resource asymmetry may constrain SMEs’ ability to respond simultaneously to economic, environmental, and social expectations.

Changes in sustainability policies have the potential to exacerbate existing workplace conflicts. SMEs may need to adjust current positions, abilities, and work methods in response to changes in manufacturing methods, technology, and organizational structures. Concerns about job security and working conditions may arise as a result of larger sustainability transitions, while Carbery & Garavan (2005) stress the importance of managing organizational change and employee development. How these changes are handled during transitions will determine their impact on social cohesiveness. Trust and inclusion in the workplace could take a hit if workers see sustainability-related reorganization as an attack on their jobs or working conditions. On the flip side, sustainability projects have the potential to increase engagement and employee dedication when they involve employees in the change and provide them with chances to learn new skills. Therefore, social cohesiveness and sustainability are not inherently at odds; rather, the nature of the link between the two is contingent upon the methods used to bring about organizational transformation.

Further, sustainability in SMEs can pose challenges to social cohesion for several reasons, such as economic limitations that restrict social investments, as most SMEs operate with low profit margins, hindering their ability to engage in sustainable practices ( Kaousar Nassr & Wehinger, 2016; Santos, 2011). SMEs’ position within wider supply chains introduces another dimension of the sustainability challenge. Many SMEs are subject to increasing expectations from customers, suppliers, regulators, and larger business partners to comply with sustainability standards ( Huq & Stevenson, 2020; Zaridis et al., 2021). Obedience may surge operational and production costs, specifically where SMEs lack the negotiating power or resources available to larger organizations. The distribution of these costs may have social implications when firms respond by increasing prices, reducing labor costs, or modifying employment conditions. However, the relationship between sustainability requirements and inequality is context-dependent, and the reviewed literature does not establish that compliance necessarily produces adverse social outcomes ( Kaymak & Poschke, 2016; Stantcheva, 2022). Rather, it emphasizes the prominence of considering how the costs and benefits of sustainability transitions are dispersed amongst firms, employees, consumers, and other stakeholders.

Another factor that affects how long SME projects last is stakeholder involvement. When stakeholders believe that their interests have not been sufficiently taken into account, they may be less likely to support sustainability policies that do not include employees, consumers, suppliers, or local communities ( Eikelenboom & Long, 2023). Conversely, transparent communication and inclusive participation may strengthen stakeholder trust and facilitate collective support for sustainability initiatives ( Tsang et al., 2009). This finding establishes a causal relationship between sustainability and the stakeholder engagement strategy put forward in this research. Small and medium-sized enterprises (SMEs) may benefit from meaningful involvement to better understand stakeholder concerns, balance competing priorities, and create sustainable strategies that are more likely to be sustained. Another factor that can affect SMEs’ capacity to implement sustainable practices is the state of regulations and institutions. There may be an imbalance in the compliance difficulties experienced by SMEs due to inconsistent, complicated, or demanding regulatory requirements, especially if these requirements are low in administrative and technical capabilities ( Favourate, 2021; John, 2024; Mallett et al., 2019; Nieuwenhuizen, 2019).

The literature as a whole suggests that small and medium-sized enterprises (SMEs) face sustainability challenges when trying to balance their social, environmental, and economic responsibilities over time. SMEs face a double-edged sword: whether they want to help bring people together, and whether they have the means, skills, support from key stakeholders, and favorable institutional environments to keep going strong while doing so. Consequently, sustainability is interdependent on the three processes outlined in the framework. Transitions to sustainability in employment practices may affect worker engagement, job stability, and job quality. According to the principle of community embeddedness, SMEs must be able to sustain their presence and contributions to communities for locally-oriented initiatives to be viable in the long run. To keep stakeholder support and meet conflicting sustainability requirements, SMEs can benefit from open and inclusive participation within stakeholder engagement. Importantly, sustainability may strengthen or constrain earlier recognized prospects. SMEs with strong community and stakeholder networks may be better able to mobilize support and knowledge for sustainability projects, reducing resource restrictions. In contrast, sustainability standards that surpass SMEs’ financial or managerial competence may hinder investment in employment, community projects, or stakeholder engagement. SMEs’ contribution to social cohesion is a dynamic process that balances economic viability, environmental responsibility, social objectives, and stakeholder expectations, not a uniformly positive outcome of sustainability activities.

### 4.5 Scalability

Finally, SMEs face challenges when it comes to taking community and social activities that have been successful and expanding them outside their initial organizational or geographical setting. When SMEs have limited resources (financial, human, managerial, and institutional), it might be challenging for them to spread their innovative and locally successful answers to social needs. Because of this, there is a significant conflict in how SMEs promote social cohesiveness: while an initiative may work wonders in one community due to its tailor-made nature, it may be difficult to replicate in another. To be scalable, an effort must be able to grow in scope while maintaining the social value it created in its original setting. This means testing if the initiative’s core techniques can be replicated elsewhere ( Gamage et al., 2020; Thorpe et al., 2005). Scaling community-oriented initiatives is impeded, according to the literature, by contextual variance due to differences in social structures, cultural expectations, resources, and institutional arrangements; an intervention that successfully tackles the needs, connections, and institutional conditions of one community could not have the same results in another ( Forrest & Wiek, 2014). Scalability poses a significant challenge for SMEs in promoting social cohesion, as their limited reach, resources, and institutional support restrict their ability to achieve large-scale transformation ( Islam, 2022; Osano, 2023). While scalability is important, it doesn’t mean small and medium-sized enterprises (SMEs) have to replicate their projects on their own or grow to the same size as big companies. Another approach for SMEs to increase the reach of locally established processes is through partnerships. Small and medium-sized enterprises (SMEs) can gain access to complementary resources, expertise, networks, and implementation capabilities through collaboration with larger organizations, governmental institutions, NGOs, and other SMEs ( Coleman et al., 2016). Scalability, according to the research, necessitates a compromise between local specificity and wider reach. While SME efforts may be more responsive and relevant to certain communities due to their highly localized nature, this same localization might make it harder to standardize and replicate. While this doesn’t mean SMEs can’t make a difference in society, it does suggest that their impact is more likely to be felt through partnerships, cumulative local effects, and the spread of practices than through massive company-wide changes. So, instead of focusing on whether individual SMEs can independently achieve large-scale effect, it may be more relevant to measure the extent to which locally successful techniques may be integrated, adapted, and shared throughout communities as a whole ( Davenport & Bibby, 1999; Purnamasari, 2024).

Taken together, the five challenges identified in this section should not be viewed as independent barriers. Limited resources can restrict SMEs’ ability to initiate and sustain social initiatives; limited reach can constrain the breadth of their influence; reconciling varied interests can complicate collective decision-making; sustainability can challenge the continuity of social contributions; and scalability can limit the transfer of successful local practices to broader contexts. The opportunities mentioned can interact with these difficulties. Partnerships enabled by well-established community ties may help offset scarce funding, stakeholder involvement may bring disparate interests into harmony, and creative or adaptable methods may ease scaling-up efforts. Consequently, the relationship that emerges is not predetermined: the interplay between SMEs’ organizational capacities, stakeholder relationships, local contexts, and the larger institutional environment determines the potential contribution of SMEs to social cohesion.

Figure 2 illustrates the aforementioned challenges that SMEs must overcome to enhance social cohesion.

**Figure 2.**
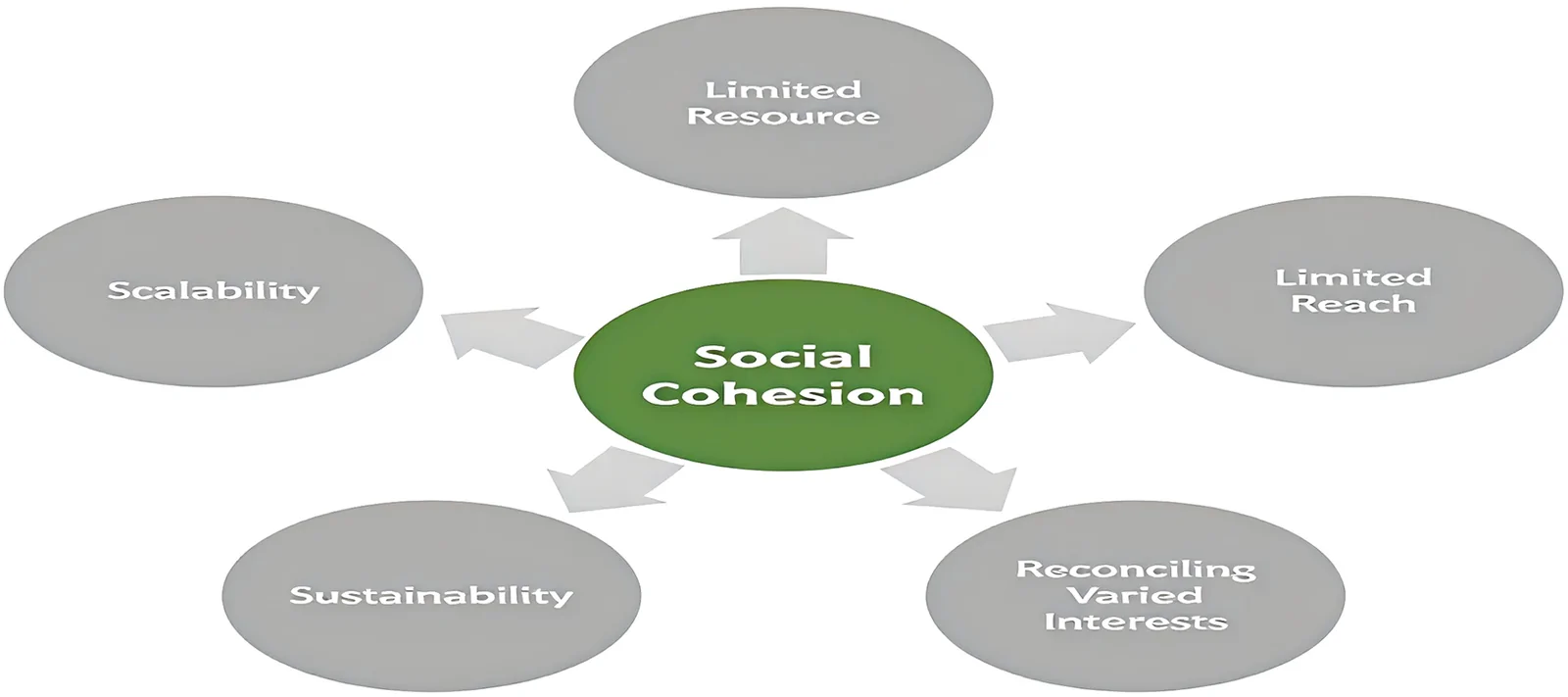
SMEs’ Five challenges that hinder social cohesion.

## 5. Conclusion

The current paper is a preliminary attempt to explore and comprehensively deliberate on the opportunities that might benefit SMEs in fostering social cohesion. However, SMEs face challenges that might hinder their efforts to foster social cohesion. This comprehensive view has the potential to provide a better understanding of the ambiguity surrounding this topic, which in turn helps SMEs boost their efforts to enhance social cohesion in two ways: first, by better exploiting the available opportunities that translate efforts into an enhanced level of social cohesion. Second, SMEs must overcome the expected challenges that might hinder their efforts to support social cohesion.

Basically, our article mainly reviews major previous work from management and economics, as well as sociological perspectives. Then, we tried to bridge them to highlight the opportunities and challenges that SMEs should take into account in their efforts to enhance social cohesion. Primarily, five factors are identified as opportunities that SMEs should consider to foster social cohesion: innovation and creativity, flexibility and agility, robust community connections, localized impact, and customized engagement. In contrast, five significant challenges have been identified that SMEs should overcome in their attempts to foster social cohesion: Limited resources, limited reach, reconciling varied interests, sustainability, and scalability. Based on an in-depth and extensive literature review, the opportunities and challenges SMEs might face in their endeavor to enhance social cohesion are represented in Figure 3.

**Figure 3.**
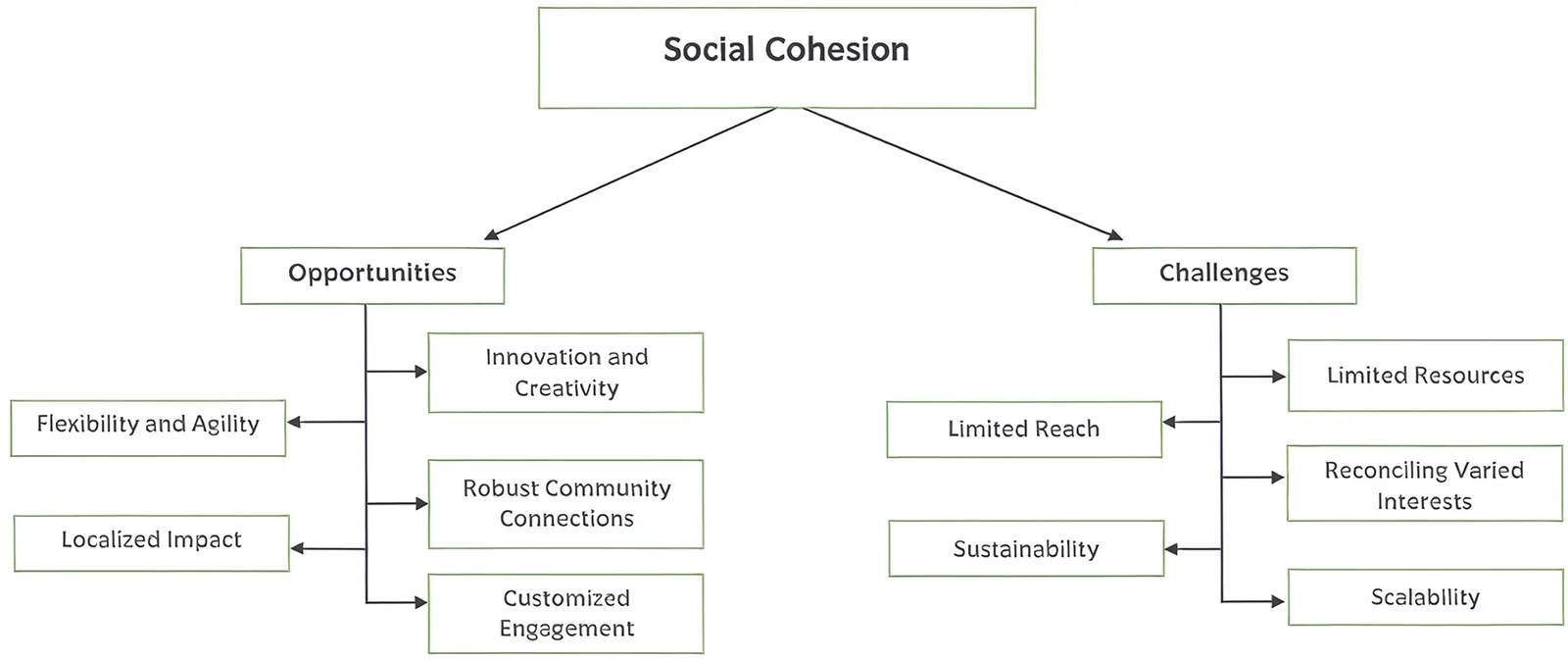
A summary of opportunities and challenges for SMEs toward social cohesion.

## 6. Discussion

This conceptual assessment identifies several interrelated organizational processes that allow SMEs to contribute to social cohesion. The literature review suggests SMEs are better equipped to foster trust, social inclusion, participation, and shared identity in their communities when they adopt community-oriented innovation, local embeddedness, stakeholder relationships, and job creation in an integrated manner. Inclusive employment practices, in particular, can enhance economic participation and reduce social exclusion; SMEs are more responsive to community needs than many larger organizations because of their close integration with local communities ( DiBella et al., 2023). The same is true of stakeholder relationships, which improve social networks and allow collaborative problem-solving by facilitating collaboration across enterprises, governmental institutions, and civil society. Community-oriented innovation raises this process to yet higher levels by allowing SMEs to create solutions tailored to their unique context ( Dewi et al., 2026).

The literature also warns against over-interpretation of these positive contributions. The ability of SMEs to create social cohesiveness is not universal. It depends on how organizational capacities interact with the institutional, economic, and social contexts of SMEs ( Dewi et al., 2026). SMEs may not be able to provide fair employment or participate in long-term community activities due to resource constraints, financial limits, managerial capacity and technological availability ( Croucher et al., 2013). Market penetration and organizational scale may limit the social impact of successful local projects. These restrictions demonstrate that even when social benefits are available for SMEs, their contribution to social cohesion is often limited by external factors ( Desa & Koch, 2014).

Moreover, the assessment reveals that the key opportunities and challenges identified in the research are not isolated but are closely interconnected. Through rapid adaptability, collaborative problem-solving, and effective use of local knowledge and networks, tight stakeholder ties and organizational flexibility of SMEs can help to compensate for resource restrictions ( Capó-Vicedo et al., 2026). Embedding in the community can compensate for some institutional or budgetary shortcomings, increasing legitimacy, trust and access to informal support systems ( Srirejeki & Khairurrizqo, 2025). On the other hand, SMEs may find it difficult to maintain community participation or to scale up successful social projects due to sustainability issues, conflicting stakeholder interests and chronic resource limitations, which might reduce the effectiveness of these benefits (Dana et al., 2021). Hence, it is important to think of the linkages between SMEs and social cohesion as fluid, with opportunities and threats feeding off each other.

The proposed conceptual framework organizes the five opportunities into three higher-order SME mechanisms: Employment Practices, Community Embeddedness, and Stakeholder Engagement. Inclusive recruitment, employee engagement, organizational flexibility and workplace innovation are employment practices that promote trust, engagement and social inclusion in organizations and their communities. Stakeholder Engagement enables SMEs to partner with consumers, suppliers, government agencies, civil society organizations, and local communities to share information, make decisions, and innovate. These techniques are complementary and can help SMEs to promote social cohesiveness in the community. The proposed framework theoretically contributes to the extant research by embedding the insights of stakeholder theory, social capital theory and the community embeddedness literature in a holistic conceptual model. Stakeholder theory highlights the importance of maintaining mutually beneficial relationships among various stakeholder groups. In contrast, social capital theory focuses on the construction of trust, reciprocity, and cooperative networks as the basis for cohesive communities. Also, the notion of community embeddedness helps to explain why SMEs are often well positioned to identify local needs and mobilize community resources. This study synthesizes these complementary perspectives to offer a more holistic account of the organizational processes that can facilitate SMEs’ influence on social cohesion.

However, the results of this review should be considered in the context of the limitations of conceptual research. While the proposed framework integrates evidence from the existing literature, it does not empirically establish causal relationships between the constructs identified. Furthermore, the strength and direction of these relationships will likely differ across institutional environments, cultural settings, industry sectors and stages of SME development. Thus, the framework should be viewed as a theoretically informed model that generates propositions for future empirical investigation rather than as definitive proof of causal effects. Further studies with quantitative, qualitative and longitudinal designs are needed to validate the proposed mechanisms, explore contextual moderators and assess the extent to which SMEs contribute to social cohesion under different economic and institutional conditions.

In summary, the conceptual model of the current research, which presents propositions that could have positive and negative influences on the role of SMEs in social cohesion, is shown in Figure 4 below.

**Figure 4.**
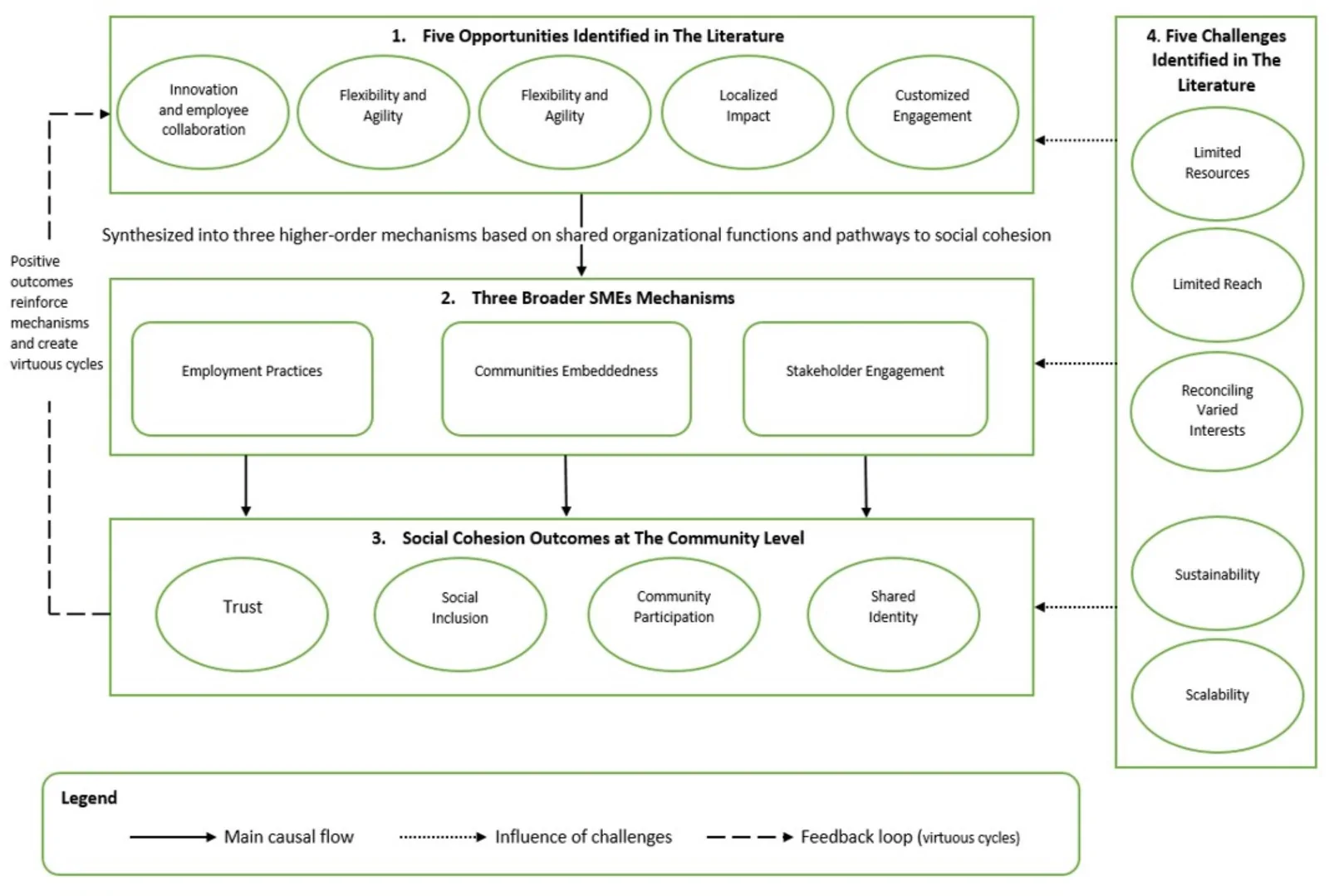
Conceptual framework.

## 7. Directions for future research

The current study considers a type of qualitative research that profoundly depends on discussion and interpretation rather than examinations using a quantitative approach that requires data and generates results through several statistical tests. Consequently, future research might focus on determining which of the aforementioned opportunities and challenges significantly impact fostering or hindering social cohesion. Likewise, a deep systematic literature review is needed for better evidence-based research among different sectors and contexts.

## Data Availability

No data are associated with this article. This study is conceptual in nature and does not involve the collection, analysis, or generation of empirical data. Therefore, no data are available.
