# Supplementary material for: SMEs as Agents of Social Cohesion: Opportunities, Challenges, and Conceptual Framework
Source: F1000Res. 2026 Oct 10;15:1045. Originally published 2026 Jun 30. [Version 2] doi: 10.12688/f1000research.183208.2 (PMC13687737; doi:10.12688/f1000research.183208.2)
Supplement: Peer review history

# Reviewer report: Reviewer response for version 2

**DOI:** 10.5256/f1000research.207808.r528637

**Published:** 2026-10-21

**Authors:** Mukhtar Mayowa Aliu

**Affiliations:** 1 Nigerian Defence Academy, Kaduna, Nigeria

I have gone through the corrected manuscript. The manuscript is satisfactory for indexing. Please accept it for indexing without any reservation.

Is the review written in accessible language?

Yes

Are all factual statements correct and adequately supported by citations?

Yes

Are the conclusions drawn appropriate in the context of the current research literature?

Partly

Is the topic of the review discussed comprehensively in the context of the current literature?

Partly

Reviewer Expertise:

NA

I confirm that I have read this submission and believe that I have an appropriate level of expertise to confirm that it is of an acceptable scientific standard.

# Reviewer report: Reviewer response for version 2

**DOI:** 10.5256/f1000research.207808.r507855

**Published:** 2026-10-21

**Authors:** Ndivhuho Tshikovhi

**Affiliations:** 1 Durban University of Technology, Durban, South Africa

The authors have revised the paper as suggested. Thus, I consider the article approved without reservations.

Is the review written in accessible language?

Yes

Are all factual statements correct and adequately supported by citations?

Partly

Are the conclusions drawn appropriate in the context of the current research literature?

Yes

Is the topic of the review discussed comprehensively in the context of the current literature?

Partly

Reviewer Expertise:

NA

I confirm that I have read this submission and believe that I have an appropriate level of expertise to confirm that it is of an acceptable scientific standard.

# Reviewer report: Reviewer response for version 1

**DOI:** 10.5256/f1000research.202227.r502123

**Published:** 2026-08-04

**Authors:** Ndivhuho Tshikovhi

**Affiliations:** 1 Durban University of Technology, Durban, South Africa

Please provide a full report, expanding on your answers to the questions above. In particular, if you answered “no” or “partly” to any of the questions, please give constructive and specific details as to how the authors can address any criticisms. Please indicate clearly which points must be addressed to make the article scientifically sound. ( *50 words min.*)

 This review addresses an important and timely topic by examining the role of SMEs in fostering social cohesion through a well-structured conceptual framework. The manuscript is clearly written, supported by relevant literature, and provides a useful synthesis of opportunities and challenges. However, the literature review would benefit from stronger critical synthesis rather than descriptive summaries. The conceptual novelty should be more clearly articulated in relation to existing frameworks, and the definition of social cohesion should be applied consistently throughout the manuscript. Additionally, greater international balance, clearer policy implications, more operational future research directions, and minor language refinements would further strengthen the manuscript. Overall, the article represents a valuable contribution and is suitable for publication after these revisions.

Is the review written in accessible language?

Yes

Are all factual statements correct and adequately supported by citations?

Partly

Are the conclusions drawn appropriate in the context of the current research literature?

Yes

Is the topic of the review discussed comprehensively in the context of the current literature?

Partly

Reviewer Expertise:

NA

I confirm that I have read this submission and believe that I have an appropriate level of expertise to confirm that it is of an acceptable scientific standard, however I have significant reservations, as outlined above.

# Response

**Published:** 2026-09-29

**Authors:** Dr malek aledenat

Comment 1:  the literature review would benefit from stronger critical synthesis rather than descriptive summaries. 

 Response: A comprehansive critical synthesisis is done 

 Comment2: The conceptual novelty should be more clearly articulated in relation to existing frameworks, and the definition of social cohesion should be applied consistently throughout the manuscript.

 Response: The conceptual novelty clearly articulated in relation to existing frameworks. In addition, the definition of social cohesion is now applied consistently throughout the manuscript

 Comment 3: A greater international balance, clearer policy implications, more operational future research directions, and minor language refinements would further strengthen the manuscript

 Response: we have done our best to consider a greater international balance, clearer policy implications, more operational future research directions. Additionaly, proofreading and editing for the whole manuscript is took a place.

# Reviewer report: Reviewer response for version 1

**DOI:** 10.5256/f1000research.202227.r499440

**Published:** 2026-07-29

**Authors:** Nicoletta Buratti

**Affiliations:** 1 University of Genoa, Genoa, Italy

The topic is relevant, and the manuscript has conceptual potential, but major revisions are required. The central construct, review method, theoretical mechanisms, and evidentiary basis of the proposed framework must be clarified before the article can be considered scientifically robust.

 The manuscript addresses a timely and potentially important topic and draws on literature from entrepreneurship, social capital, corporate social responsibility, sustainability, and community development. It is generally accessible and includes references published up to 2024. Nevertheless, the conceptual focus and internal presentation require substantial clarification. “Social cohesion” alternates between cohesion among employees inside SMEs and cohesion within the communities in which SMEs operate. These are different levels of analysis and should be explicitly defined and related.

 The abstract, main classification, and figures are not fully aligned. The abstract emphasizes job creation, social inclusion, community-driven innovation, weak institutional environments, and informality, whereas the body is organized around five different opportunities and five different challenges. Figure 4 then introduces employment practices, community embeddedness, and stakeholder engagement without explaining how these constructs were derived.

 The summary of Section 2 also refers to five opportunities but effectively names six by treating employee collaboration separately from innovation and creativity.

 The figures need legends, explicit arrow direction, and a clear correspondence with the text.

 Terminology also shifts among SMEs, social enterprises, advocacy organizations, and community organizations.

 Finally, several illustrative examples are presented as factual evidence without direct support.

 The authors should align all sections and figures, verify the relevance of each citation, engage more systematically with recent work on social-cohesion measurement and MSME social sustainability, and undertake a thorough language edit.

 As regards the Discussion section, the reviewed literature broadly supports the cautious conclusion that SMEs may contribute to social cohesion through employment, local embeddedness, stakeholder relationships, and community-oriented innovation. At the same time, resource scarcity, limited reach, conflicting interests, sustainability challenges, and scalability constraints can limit that contribution. The stronger claims are not yet adequately supported.

 The Discussion largely repeats the two lists instead of interpreting their interactions or comparing the framework with prior theory. A separate Conclusion is also absent.

 The revised Discussion should explain how opportunities may offset or intensify particular challenges, how the three proposed SME mechanisms connect to the three social-cohesion outcomes, and under which contextual conditions these relationships are expected to hold. A distinct Conclusion should summarize the contribution, state the limitations of a conceptual review, and use appropriately cautious language.

Is the review written in accessible language?

Partly

Are all factual statements correct and adequately supported by citations?

Partly

Are the conclusions drawn appropriate in the context of the current research literature?

Partly

Is the topic of the review discussed comprehensively in the context of the current literature?

Partly

Reviewer Expertise:

Business management; entrepreneurship; community enterprise

I confirm that I have read this submission and believe that I have an appropriate level of expertise to confirm that it is of an acceptable scientific standard, however I have significant reservations, as outlined above.

# Response

**Published:** 2026-08-01

**Authors:** Dr malek aledenat

We sincerely thank the reviewer for the careful evaluation of our manuscript and for providing constructive comments that have significantly improved the quality, clarity, and scholarly contribution of our work. We have carefully considered every suggestion and substantially revised the manuscript accordingly. Below, we provide a detailed point-by-point response.

**Reviewer Comment 1**

 The conceptual focus and internal presentation require substantial clarification. “Social cohesion” alternates between cohesion among employees inside SMEs and cohesion within the communities in which SMEs operate. These are different levels of analysis and should be explicitly defined and related.

**Response**

 Thank you for this valuable observation. We fully agree that the previous version did not sufficiently distinguish between organizational-level cohesion and community-level social cohesion.

 To address this concern, we substantially revised the Introduction by introducing a dedicated subsection entitled **"Conceptualizing Social Cohesion."** In this section, we clearly define **community-level social cohesion** as the principal outcome of the conceptual framework, characterized by trust, inclusion, participation, connectedness, and shared identity within communities.

 We further clarify that **employee cohesion** is not treated as the primary outcome but rather as an internal organizational mechanism that strengthens SMEs' capacity to generate broader community-level social cohesion. Throughout the manuscript, terminology has been revised to consistently distinguish these two levels of analysis and to explain their relationship within the proposed framework.

**Reviewer Comment 2**

 The abstract, main classification, and figures are not fully aligned.

**Response**

 We appreciate this important observation.

 The manuscript has been comprehensively reorganized to ensure complete consistency among the Abstract, the main body, and all conceptual figures.

 Specifically,

- the Abstract now reflects the same conceptual structure presented in the manuscript;
- the opportunities and challenges discussed throughout the text directly correspond to those illustrated in the conceptual framework;
- all figures have been redesigned to mirror the narrative developed in the manuscript.

This revision establishes a coherent conceptual flow from the Abstract through the Discussion and the proposed framework.

**Reviewer Comment 3**

 The abstract emphasizes job creation, social inclusion, community-driven innovation, weak institutional environments, and informality, whereas the body is organized around five different opportunities and five different challenges. Figure 4 then introduces employment practices, community embeddedness, and stakeholder engagement without explaining how these constructs were derived.

**Response**

 We thank the reviewer for identifying this inconsistency.

 The revised manuscript now explicitly explains that the five identified opportunities naturally converge into three broader SME mechanisms:

- **Employment Practices**
- **Community Embeddedness**
- **Stakeholder Engagement**

Each mechanism is now introduced and theoretically justified within the literature review before appearing in Figure 4. The revised conceptual framework clearly illustrates how these mechanisms emerge from the preceding discussion rather than being introduced independently.

 Furthermore, the Abstract has been revised to reflect these same mechanisms, ensuring consistency throughout the manuscript.

**Reviewer Comment 4**

 The summary of Section 2 also refers to five opportunities but effectively names six by treating employee collaboration separately from innovation and creativity.

**Response**

 We appreciate this careful observation.

 To eliminate this inconsistency, we merged **employee collaboration and teamwork** with **innovation and creativity** into a single integrated opportunity because collaboration represents one of the principal mechanisms through which innovation is achieved within SMEs.

 Consequently, Section 2 now consistently discusses **five opportunities**, and the summary, figures, and conceptual framework have all been updated accordingly.

**Reviewer Comment 5**

 The figures need legends, explicit arrow direction, and a clear correspondence with the text.

**Response**

 Thank you for this constructive recommendation.

 All conceptual figures have been completely redesigned.

 The revised figures now include:

- descriptive titles and legends,
- clearly labeled directional arrows,
- consistent terminology,
- explicit relationships among constructs,
- direct correspondence with the narrative presented in the manuscript.

Each figure is now introduced and discussed within the relevant section of the text before being presented.

**Reviewer Comment 6**

 Terminology also shifts among SMEs, social enterprises, advocacy organizations, and community organizations.

**Response**

 We agree with the reviewer that inconsistent terminology could create conceptual ambiguity.

 Throughout the revised manuscript, we consistently use **Small and Medium Enterprises (SMEs)** as the focal unit of analysis.

 References to social enterprises, advocacy organizations, or community organizations have either been removed or clearly presented as illustrative examples of particular types of SMEs where appropriate.

 This revision improves conceptual consistency across the manuscript.

**Reviewer Comment 7**

 Several illustrative examples are presented as factual evidence without direct support.

**Response**

 Thank you for highlighting this issue.

 All illustrative examples have been carefully reviewed.

 Unsupported examples have either:

- been replaced with evidence from published literature,
- been supported by appropriate references, or
- been rewritten as hypothetical illustrations using cautious language such as "for example" or "SMEs may."

This revision ensures that all empirical statements are appropriately supported by the literature.

**Reviewer Comment 8**

 The authors should align all sections and figures, verify the relevance of each citation, engage more systematically with recent work on social-cohesion measurement and MSME social sustainability, and undertake a thorough language edit.

**Response**

 We sincerely appreciate this comprehensive recommendation.

 Accordingly,

- all sections have been reorganized to improve conceptual consistency;
- all figures have been revised to correspond directly with the manuscript;
- every citation has been reviewed to ensure relevance and accuracy;
- numerous recent references (particularly from 2022–2026) addressing social cohesion, SME sustainability, stakeholder engagement, community embeddedness, and social-capital theory have been incorporated;
- the manuscript has undergone a comprehensive language revision to improve clarity, grammar, readability, and academic style.

These revisions substantially strengthen both the theoretical foundation and overall presentation of the manuscript.

**Reviewer Comment 9**

 As regards the Discussion section, the reviewed literature broadly supports the cautious conclusion that SMEs may contribute to social cohesion through employment, local embeddedness, stakeholder relationships, and community-oriented innovation. At the same time, resource scarcity, limited reach, conflicting interests, sustainability challenges, and scalability constraints can limit that contribution. The stronger claims are not yet adequately supported.

**Response**

 We appreciate this insightful observation.

 The Discussion has been completely rewritten to adopt a more balanced and evidence-based interpretation.

 Rather than making definitive causal claims, the revised manuscript now emphasizes that SMEs **have the potential** to contribute to social cohesion under favorable organizational and institutional conditions.

 The revised Discussion explicitly acknowledges that this contribution is contingent upon overcoming important organizational and contextual constraints, including limited resources, restricted outreach, conflicting stakeholder interests, sustainability challenges, and scalability limitations.

 Accordingly, all conclusions now employ appropriately cautious language consistent with the conceptual nature of the study.

**Reviewer Comment 10**

 The Discussion largely repeats the two lists instead of interpreting their interactions or comparing the framework with prior theory. A separate Conclusion is also absent.

**Response**

 We fully agree with this recommendation.

 The Discussion section has been substantially expanded to move beyond descriptive summaries.

 Specifically, it now:

- interprets the interactions between opportunities and challenges;
- compares the proposed framework with stakeholder theory, social capital theory, and community embeddedness perspectives;
- explains the theoretical contribution of integrating these perspectives within a unified conceptual model.

Additionally, a completely new **Conclusion** section has been added.

 The Conclusion summarizes the major contributions of the study, highlights theoretical and practical implications, acknowledges the limitations of conceptual research, and identifies directions for future empirical validation.

**Reviewer Comment 11**

 The revised Discussion should explain how opportunities may offset or intensify particular challenges, how the three proposed SME mechanisms connect to the three social-cohesion outcomes, and under which contextual conditions these relationships are expected to hold. A distinct Conclusion should summarize the contribution, state the limitations of a conceptual review, and use appropriately cautious language.

**Response**

 Thank you for this valuable recommendation.

 The revised Discussion now explicitly explains:

- how opportunities such as innovation, flexibility, and community embeddedness can mitigate challenges associated with limited resources, stakeholder conflicts, and scalability;
- how unfavorable institutional environments or resource scarcity may weaken these positive relationships;
- how the three proposed SME mechanisms (Employment Practices, Community Embeddedness, and Stakeholder Engagement) collectively contribute to three dimensions of social cohesion, namely trust, inclusion, and community participation;
- the contextual conditions under which these relationships are expected to operate, particularly in emerging and resource-constrained economies.

Finally, a separate Conclusion has been added that summarizes the study's contribution, discusses the inherent limitations of a conceptual review, proposes future research directions, and consistently employs cautious, evidence-based language throughout.

 We sincerely thank the reviewer for the thoughtful and constructive comments. We believe that the extensive revisions undertaken in response to these suggestions have substantially strengthened the manuscript by improving its conceptual clarity, theoretical coherence, methodological rigor, and overall scholarly contribution. We are grateful for the opportunity to revise the manuscript and hope that the revised version satisfactorily addresses all concerns raised by the reviewer.

# Reviewer report: Reviewer response for version 1

**DOI:** 10.5256/f1000research.202227.r502115

**Published:** 2026-07-23

**Authors:** Mukhtar Mayowa Aliu

**Affiliations:** 1 Nigerian Defence Academy, Kaduna, Nigeria

The topic "SMEs as Agents of Social Cohesion: Opportunities, Challenges, and Conceptual Framework" is good and conceptually sound. Nevertheless, the conceptual review is content and conceptually lacking. The framework adopted in the study is not properly discussed in line with how such adopted framework can actively used as agent for social cohesion amongst various challenges facing SMEs.

 In addition, although conclusion were partially drawn in the discussion section of the study, the discussion should be separated from the study's conclusion. The discussion of the study is not comprehensively enough. The Author(s) should further elaborate on the potential importance of adopting such a conceptual framework.

Is the review written in accessible language?

Yes

Are all factual statements correct and adequately supported by citations?

Yes

Are the conclusions drawn appropriate in the context of the current research literature?

Partly

Is the topic of the review discussed comprehensively in the context of the current literature?

Partly

Reviewer Expertise:

Entrepreneurship, management and business studies

I confirm that I have read this submission and believe that I have an appropriate level of expertise to confirm that it is of an acceptable scientific standard, however I have significant reservations, as outlined above.

# Response

**Published:** 2026-08-01

**Authors:** Dr malek aledenat

Reviewer Comment 1

 The framework adopted in the study is not properly discussed in line with how such adopted framework can actively used as agent for social cohesion amongst various challenges facing SMEs.

 Response: An adequate discussion was added.

 Reviewer Comment 2

 In addition, although conclusion were partially drawn in the discussion section of the study, the discussion should be separated from the study's conclusion.

 Response: Done.

 Reviewer Comment 3

 The discussion of the study is not comprehensively enough. The Author(s) should further elaborate on the potential importance of adopting such a conceptual framework.

 Response: Done.

 We sincerely thank the reviewer for the thoughtful and constructive comments. We believe that the extensive revisions undertaken in response to these suggestions have substantially strengthened the manuscript by improving its conceptual clarity, theoretical coherence, methodological rigor, and overall scholarly contribution. We are grateful for the opportunity to revise the manuscript and hope that the revised version satisfactorily addresses all concerns raised by the reviewer.
